# Supplementary material for: General practitioner workforce sustainability to maximise effective and equitable patient care: a realist review protocol
Source: BMJ Open. 2024 May 21;14(5):e075189. doi: 10.1136/bmjopen-2023-075189 (PMC11110576; doi:10.1136/bmjopen-2023-075189)
Supplement: Supplementary data [file bmjopen-2023-075189supp001.pdf]

Supplementary File 1

Details of the search developed for MEDLINE:

**Data Source**	Database	MEDLINE	
	Details	MEDLINE® Epub Ahead of Print, In-Process & Other Non-Indexed Citations, Ovid MEDLINE® Daily and Ovid MEDLINE® 1946 to present	
	Host	Ovid	
	Date searched	20/04/2023	
	Database Update	Daily update	
**Limiters**	English only?	Yes	
	Time period searched	2013 - Present	
	Publications types	No limit	
	Other		
**Results**	Items found	504	
	Internal duplicates (within one database)	6	
	External duplicates (between databases)	0	
	New	498	
In Ovid, save your search. Go to **Saved Searches** and click on the eye icon of your SR saved search to **View** your search. Copy and paste your search into **Notepad;** put tabs between the line number and text. Copy and paste into cell **B15**.	**Paste text of search strategy below**		
	**Saved: Sustainable GP V6 FINAL MEDLINE**		
	Medline (Ovid MEDLINE® Epub Ahead of Print, In-Process & Other Non-Indexed Citations, Ovid MEDLINE® Daily and Ovid MEDLINE®) 1946 to present		
	1	general practi*.ti,ab,kw.	92437
	2	*General Practitioners/	8488
	3	exp *General Practice/	51683
	4	or/1-3	121611
	5	exp *Work/	43272
	6	exp *Workplace/	14,019
	7	*Job Satisfaction/	12818
	8	exp *Occupational Stress/	16249
	9	exp *"Personnel Staffing and Scheduling"/	24174
	10	(work* or staffing or career* or job*).ti,ab,kw.	2041372
	11	(stress* or burn out or burnout).ti,ab,kw.	1066414
	12	or/5-11	3013398
	13	exp *Workforce/	7820
	14	*Retirement/	6784
	15	*Personnel Turnover/	3003
	16	*Work Engagement/	559
	17	*Career Mobility/	6434
	18	(workforce* or staff* level*).ti,ab,kw.	37788
	19	(part?time* or "less than full time" or LTFT).ti,ab,kw.	239
	20	(retire* or retention* or retain* or sustain* or stability or turnover).ti,ab,kw.	1509240
	21	(work* adj1 pattern*).ti,ab,kw.	1433

		22	(flexible* adj1 work*).ti,ab,kw.	1028
		23	((reason* or rationale* or decision* or intention*) adj1 (leav* or stay*)).ti,ab,kw.	313
		24	(career* adj1 (break* or end*)).ti,ab,kw.	312
		25	((participat* or engag*) adj1 (work* or job*)).ti,ab,kw.	6597
		26	or/13-25	1560462
		27	4 and 12 and 26	2544
		28	exp United Kingdom/	389181
		29	(national health service* or nhs*).ti,ab,in.	265557
		30	(english not ((published or publication* or translat* or written or language* or speak* or literature* or citation*) adj5 english)).ti,ab.	48488
		31	(gb or "g.b." or britain* or (british* not "british columbia") or uk or "u.k." or united kingdom* or (england* not "new england") or northern ireland* or northern irish* or scotland* or scottish* or ((wales or "south wales") not "new south wales") or welsh*).ti,ab,jw,in.	2421175
		32	(bath or "bath's" or ((birmingham not alabama*) or ("birmingham's" not alabama*) or bradford or "bradford's" or brighton or "brighton's" or bristol or "bristol's" or carlisle* or "carlisle's" or (cambridge not (massachusetts* or boston* or harvard*)) or ("cambridge's" not (massachusetts* or boston* or harvard*)) or (canterbury not zealand*) or ("canterbury's" not zealand*) or chelmsford or "chelmsford's" or chester or "chester's" or chichester or "chichester's" or coventry or "coventry's" or derby or "derby's" or (durham not (carolina* or nc)) or ("durham's" not (carolina* or nc)) or ely or "ely's" or exeter or "exeter's" or gloucester or "gloucester's" or hereford or "hereford's" or hull or "hull's" or lancaster or "lancaster's" or leeds* or leicester or "leicester's" or (lincoln not nebraska*) or ("lincoln's" not nebraska*) or (liverpool not (new south wales* or nsw)) or ("liverpool's" not (new south wales* or nsw)) or ((london not (ontario* or ont or toronto*)) or ("london's" not (ontario* or ont or toronto)) or manchester or "manchester's" or (newcastle not (new south wales* or nsw)) or ("newcastle's" not (new south wales* or nsw)) or norwich or "norwich's" or nottingham or "nottingham's" or oxford or "oxford's" or peterborough or "peterborough's" or plymouth or "plymouth's" or portsmouth or "portsmouth's" or preston or "preston's" or ripon or "ripon's" or salford or "salford's" or salisbury or "salisbury's" or sheffield or "sheffield's" or southampton or "southampton's" or st ablans or stoke or "stoke's" or sunderland or "sunderland's" or truro or "truro's" or wakefield or "wakefield's" or wells or westminster or "westminster's" or winchester or "winchester's" or wolverhampton or "wolverhampton's" or (worcester not (massachusetts* or boston* or harvard*)) or ("worcester's" not (massachusetts* or boston* or harvard*)) or (york not ("new york" or ny or ontario* or ont or toronto*)) or ("york's" not ("new york" or ny or ontario* or ont or toronto*))))).ti,ab,in.	1720005
		33	(bangor* or "bangor's" or cardiff or "cardiff's" or newport or "newport's" or st asaph or "st asaph's" or st davids or swansea or "swansea's").ti,ab,in.	69203
		34	(aberdeen or "aberdeen's" or dundee or "dundee's" or edinburgh or "edinburgh's" or glasgow or "glasgow's" or inverness or (perth not australia*) or	253603
		35	"newry's").ti,ab,in.	33231
		36	or/28-35	3037247
		37	(exp africa/ or exp americas/ or exp antarctic regions/ or exp arctic regions/ or exp asia/ or exp oceania/) not (exp United Kingdom/ or europe/)	3308914
		38	36 not 37	2875391
		39	27 and 38	793
		40	limit 39 to (english language and yr="2013 -Current")	504
