# Supplementary material for: General practitioner workforce sustainability to maximise effective and equitable patient care: a realist review protocol
Source: BMJ Open. 2024 May 21;14(5):e075189. doi: 10.1136/bmjopen-2023-075189 (PMC11110576; doi:10.1136/bmjopen-2023-075189)
Supplement: Author's manuscript [file bmjopen-2023-075189.draft_revisions.pdf]

BMJ Open is committed to open peer review. As part of this commitment we make the peer review history of every article we publish publicly available.

When an article is published we post the peer reviewers' comments and the authors' responses online. We also post the versions of the paper that were used during peer review. These are the versions that the peer review comments apply to.

The versions of the paper that follow are the versions that were submitted during the peer review process. They are not the versions of record or the final published versions. They should not be cited or distributed as the published version of this manuscript.

BMJ Open is an open access journal and the full, final, typeset and author-corrected version of record of the manuscript is available on our site with no access controls, subscription charges or pay-per-view fees (http://bmjopen.bmj.com).

If you have any questions on BMJ Open's open peer review process please email info.bmjopen@bmj.com

BMJ Open

# General practitioner workforce sustainability to maximise effective and equitable patient care: a realist review protocol.

| | |
|---|---|
| Journal: | *BMJ Open* |
| Manuscript ID | bmjopen-2023-075189 |
| Article Type: | Protocol |
| Date Submitted by the Author: | 28-Apr-2023 |
| Complete List of Authors: | Park, Sophie; University College London, Department of Primary Care and Population Health<br>Owen, Emily; University College London, Department of Primary Care & Population Health<br>Duddy, Claire; University of Oxford, Nuffield Department of Primary Care Health Sciences<br>Rapley, Tim; Northumbria University, Department of Social Work, Education and Community Wellbeing<br>Goodman, Claire; University of Hertfordshire, Centre for Research in Public Health and Community Care<br>Cohen, Tanya; University College London, Department of Primary Care and Population Health<br>Henry , Cecily ; University College London, Department of Primary Care and Population Health<br>Ogden , Margaret; University College London, Department of Primary Care and Population Health<br>Rees, Eliot; University College London, Department of Primary Care and Population Health; Keele University, School of Medicine<br>Burford, Bryan; Newcastle University, School of Medicine<br>Lamb , Elizabeth; Newcastle University, School of Medicine<br>Vance, Gillian ; Newcastle University, School of Medicine |
| Keywords: | Health Equity, Organisation of health services < HEALTH SERVICES ADMINISTRATION & MANAGEMENT, Patient-Centered Care, Physicians, Primary Care < Primary Health Care |
| | |

SCHOLARONE™
Manuscripts

RUNNING HEAD: REALIST REVIEW PROTOCOL

# General practitioner workforce sustainability to maximise effective and equitable patient care: a realist review protocol.

Sophie Park[1], Emily C. Owen[1], Claire Duddy[2], Tim Rapley[3], Claire Goodman[4], Tanya Cohen[1], Cecily Henry[1], Margaret Ogden[1], Eliot L. Rees[1,5], Bryan Burford[6], Elizabeth I. Lamb[6], Gillian Vance[6].

[1]Department of Primary Care and Population Health, University College London, London, UK, [2]Nuffield Department of Primary Care Health Sciences, University of Oxford, Oxford, UK, [3]Department of Social Work, Education and Community Wellbeing, Northumbria University, Newcastle Upon Tyne, UK [4]Centre for Research in Public Health and Community Care, University of Hertfordshire, Hatfield, UK, [5]School of Medicine, Keele University, Keele, UK, [6]School of Medicine, Newcastle University, Newcastle Upon Tyne, UK.

Corresponding author:

Professor Sophie Park; UCL Research Department of Primary Care & Population Health; Royal Free Campus; Hampstead, London; NW3 2PY.

Email: sophie.park@ucl.ac.uk; Phone: 020 7794 0500.

Keywords: General Practice; Workforce; Sustainability; Healthcare system; Retention.

Word count: 5275.

For peer review only

ABSTRACT

**Introduction:** There are not enough General Practitioners (GPs) in the UK National Health Service. This problem is worse in areas of the country where poverty and underinvestment in health and social care mean patients experience poorer health compared with wealthier regions. Encouraging more doctors to choose and continue in a GP career is a government priority. This review will examine which aspects of the healthcare system affect GP workforce sustainability, how, why, and for whom.

**Methods and analysis:** A realist review is a theory-driven interpretive approach to evidence synthesis, that brings together qualitative, quantitative, mixed-methods research, and grey literature. We will use a realist approach to synthesise data from the available published literature to refine an evidence-based programme theory that will identify the important contextual factors and underlying mechanisms that underpin observed outcomes relating to GP workforce sustainability. Our review will follow Pawson's five iterative stages: (1) finding existing theories; (2) searching for evidence; (3) article selection; (4) data extraction and (5) synthesising evidence and drawing conclusions. We will work closely with key stakeholders and embed patient and public involvement throughout the review process to refine the focus of the review and enhance the impact and relevance of our research.

**Ethics and dissemination:** This review does not require formal ethical approval as it draws on secondary data from published articles and grey literature. Findings will be disseminated through multiple channels, including publication in peer-reviewed journals, at national and

For peer review only

international conferences, and other digital scholarly communication tools such as video summaries, Twitter, and blog posts.

**PROSPERO registration number**: CRD42023395583.

Strengths and limitations of this study:

- This is the first realist review to explore which aspects of the healthcare system affect GP workforce sustainability, how, why, and for whom.
- Our findings will offer structural and organisational recommendations to inform sustainable, context-specific ways of future working, underpinning effective and equitable patient care.
- We will embed public co-applicants and stakeholders throughout the design, analysis, and report stages of the project, enhancing the impact and relevance of our research.
- We will only include literature that is written or translated in English.

For peer review only

## INTRODUCTION

UK general practice has been described as being in crisis (1). A shortage and exodus (2) of General Practitioners (GPs) is an urgent and challenging problem, attracting significant media attention and widespread public debate. The National Health Service (NHS) Long Term Plan proposed a net increase of 5000 GPs, alongside expectations of work distribution and re-allocation across primary care teams (3). The impact of these changes on the GP workforce remains relatively unexplored.

It is widely acknowledged that general practice provides 90% of patient care utilising approximately 8% of the NHS budget (4), meaning this is a key element of broader debates about a whole-system NHS moral and financial crisis. Existing research has focused on 'numbers in' 'numbers out' calculations of workforce need, predictions, and requirements. GPs' decisions to stay or leave are often positioned as an individual 'choice' (5, 6, 7), resilience or failure, with less attention given to the social and organisational milieu in which those decisions are produced. A systems approach recognises the need to acknowledge this inherent dynamic complexity, by studying the interconnected components (e.g. the nature of social interactions between patients, colleagues, and external institutions, alignment of personal and policy priorities, organisational support structures, and leadership culture) that work together in an integrated and coordinated way to sustain and enhance the GP workforce.

The figures reflecting shifts in the number of GPs available are well-rehearsed. For example, as of February 2023, the number of full-time equivalent GPs has fallen significantly (approximately 2,100 fewer fully qualified full-time GPs compared to September 2015) (8), and UK GPs have reported higher levels of emotional distress than GPs in nearly all other countries (9). In parallel, readily measurable elements of GP work have been reported to increase, with 10.9% more GP consultations in 2021 compared with 2019, and a 10%

For peer review only

increase in practice list size (28). Consultations are reported to have increased in length (10) alongside changes in form (e.g. use of technology). Measures of GP time spent on operational challenges and problems are high (11) and the 'hidden', non-patient-facing work of GPs is increasingly being recognised (12). It is widely acknowledged that GPs staying in practice has benefits for both patient care (13, 14) and practice productivity (15). For example, experienced GPs work more efficiently, lessening the scale of patient medicalisation, investigation, and treatment, and requiring shorter appointment times compared to trainees and other healthcare professionals (16, 17, 18). We can also see that practices with a higher proportion of patients in deprived areas; lower care quality commission ratings; single-handed and rural practices, have fewer GPs overall and more locums (19) emphasising the importance of wider contextual factors in shaping the nature of work and workforce patterns (20).

A systems approach is well suited as a basis for examining the multitude of factors influencing the GP workforce and how these ultimately shape career decisions. By taking a system and theory-driven approach to understanding what works, in which contextual circumstances, and to what extent, we aim to explore and make visible the often unsaid or implicit issues that influence working environments, relationships, and organisational culture. The findings from our review will offer structural and organisational recommendations to inform sustainable, context-specific ways of future working, which can underpin effective and equitable patient care.

## METHODS

### Review aim, questions, and objectives

For peer review only

**Aim**

This review aims to examine which aspects of the healthcare system affect GP workforce sustainability, how, why and for whom.

**Research Objectives:**

1. Develop a programme theory through an evidence synthesis of how the GP workforce can be sustainable and work effectively with others to support effective and equitable patient care.
2. Embed and respond to patient and public involvement (PPI) and stakeholder perspectives throughout the design, analysis and report stages of the project, thus maximising the relevance and utility of review findings.
3. Make recommendations for practice and policy based on the refined programme theory.

**Research Questions:**

What can we learn from the existing literature that will promote GP workforce sustainability, to support effective and equitable patient care? Specifically:

1. Which mechanisms produce intended and/or unintended outcomes in GP workforce sustainability?

For peer review only

2. Which contexts influence whether different mechanisms produce intended and/or unintended outcomes in GP workforce sustainability?

3. Which interventional strategies lead to intended and/or unintended outcomes for GP workforce sustainability?

Approach

We will conduct a realist review, synthesising data from the published literature to produce a refined evidence-based programme theory that will identify the important contexts and mechanisms which underpin observed outcomes relating to GP workforce sustainability to support the delivery of effective and equitable patient care. A realist review is an interpretive and theory-driven approach to evidence synthesis, that brings together data drawn from quantitative, qualitative, and mixed methods research, as well as the wider grey literature (21). Using this data and a realist logic of analysis will allow us to examine diverse evidence with a clear focus on understanding factors which support or challenge GP workforce sustainability, how these are shaped by important contexts, and the mechanisms that underpin them. Based on this understanding, captured in our final programme theory, we will be able to identify and prioritise important system-level contexts that may be amenable to change.

Initial scoping searches using EMBASE, MEDLINE and PsycINFO identified a range of qualitative and quantitative primary and secondary evidence focused on GP retention. We have identified four existing reviews focused on GP retention (6, 22, 23, 24), and four additional studies from broader scoping searches, including one examining factors associated with part-time working (25). Existing studies on this topic are often descriptive, identifying a complex array of factors associated with workforce attrition without theoretical analysis.

For peer review only

These include individual issues (e.g. wellbeing, burnout, identity), practice (e.g. workload, administrative burden, clinical autonomy (22) - and opportunities for continuity of care (23) (6), and national (e.g. revalidation) contexts (1, 26, 27, 28). One study found the intrinsic attractions of retirement (e.g. increased time for leisure') contributed to GPs leaving practice (29). Factors shaping GP retention in one UK region (30) were described as 'push' (workload, stress, job dissatisfaction, and organisational injustice de-motivating factors) and 'pull' (work-life balance).

This review will attend to the complex systems in which GPs work, in order to better understand the factors which, keep, sustain and enable GPs to flourish within their work. A realist approach will allow us to examine a wide range of literature about the system and related social interactions (e.g. across the primary and secondary care interface, between patients and clinicians), and to integrate patient and stakeholder experience to inform our approach and produce new ways of thinking about this challenging field. We will identify causal explanations to illuminate the connections between GP work (e.g. nature and scope) and GP career intentions. Depending on the available evidence, we aim to examine a range of factors and circumstances (e.g. location, employment arrangements such as small or large institutions, salaried, partnered, payment by performance), systems of support inside or outside practices and patient deprivation. We will examine the relationships between these contextual factors and a range of reported outcomes. This will help to identify the circumstances in which a sustainable and flourishing GP workforce for delivery of effective and equitable patient care can be realised.

Our approach will follow Pawson's (21) five iterative stages for realist reviews detailed below. This begins with an initial programme theory developed through scoping and preliminary consultation with stakeholders and patients. As the review progresses, we will

For peer review only

gather and interpret data from the literature to refine and develop our explanations of important outcomes, drawing on the expertise of our PPI and stakeholder groups. At the end of the project, our final programme theory will consist of evidenced context-mechanism-outcome configurations (CMOCs), presenting realist causal explanations for outcomes relating to the GP workforce sustainability to support effective and equitable patient care.

**Patient and Public Involvement**

We have three PPI co-applicants within our core research team. The team also includes professionals from a range of disciplinary backgrounds including general practice, secondary care, nursing, psychology, and sociology. Additionally, we have recruited six PPI co-applicants from coastal, rural, and diverse urban contexts. An equality of opportunity approach will be taken throughout this process, enabling us to respond in an agile way to maximise PPI involvement. For example, helping to refine the programme theory, shape our findings and contribute to both the format and content of our outputs (e.g., written word, infographics), and ensuring that proposed solutions are both feasible and acceptable. Finally, our PPI members will play an essential role in developing our dissemination strategy, and the format and content of the outputs of this project. We also have a stakeholder group including professionals representing a variety of GP employment models, paramedic, pharmacy, policy, practice, management, and educational backgrounds.

In the development of this proposal, we invited PPI and stakeholders to share their priorities. GPs often work at the interface between human experience and the technicalities of pathology, negotiating boundaries and overlaps between stress, illness and disease (31). Person-centred care has been recognised as a key element of this process (32). Knowledge of the ‘person’ can be established over time, building on trust and relational currency between patient and practitioner (33). One key area of concern in PPI and stakeholder discussions

For peer review only

during development of this review protocol, was ‘care of other’. GPs spoke about the importance of person-centred care, while PPI co-applicants highlighted the importance of looking after practitioners and supporting their well-being. As one public contributor explained, if patients are treated as ‘a number’ within the healthcare system, this is potentially mirrored in their treatment and expectation of healthcare professionals. One key area about GP workforce sustainability in this review, therefore, is the relational elements of GP work, and how these may support or undermine workforce sustainability.

We invited PPI and stakeholders to help produce three case study examples about GP workforce sustainability:

**Case Study 1.** Multi-disciplinary team working in general practice is increasing in response to the NHS Long Term Plan (3), with many practices now employing pharmacists, paramedics, physicians associates, physiotherapists and mental health professionals. In some circumstances, this has enabled GPs to focus on the clinical care of particularly ‘complex’ patients (e.g. with multimorbidity), delegate some home visits (34), and prescribing demands (35). Yet, some stakeholders report a) anecdotal increases in workload relating to supervision and support of new staff and b) increased levels of follow up (e.g. lower thresholds for initiating patient investigations), c) patients needing to attend multiple appointments to resolve a problem(s) and d) challenges negotiating access for patients with unselected and comprehensive healthcare problems to see the most appropriate professional, at the most appropriate time. These fast-moving changes to the GP role and patient access to GP care, have therefore been experienced both as producing opportunities and challenges.

**Case Study 2.** Relationships between GPs and secondary care colleagues can influence patient care and access to services. For example, if close and rapid communication is established (e.g. informal sharing of numbers, or formal routes for email advice and

For peer review only

guidance), GPs can progress patient care, minimising referrals and waiting list delays, and maximising timely patient care delivery in the primary care setting. This could have an impact on a GP's sense of agency or autonomy to provide immediate care for patients, but also a sense of working as part of a distributed team, rather than alone. As an example: a specialist nurse managing a Paediatric Allergy clinic advice service. In one area, this enables access to advice re: initial investigations and treatment. In another area, this increases recommendations to refer. This means waiting lists could be shortened or lengthened, expediting or delaying assessment of urgent or severe patients, or periods of time requiring additional GP support.

**Case Study 3.** GPs sometimes develop 'special interests' (e.g. supporting patients with mental health problems). This can be positive for practices, patients, and GP professional development. Yet, it can also produce challenges and inequalities if not managed well. For example, patients requiring more clinical input, longer consultations and emotional investment being directed to seek help from particular GPs. Without adequate planning, this can result in GPs who support these patients running late, working longer hours (for no extra pay) to complete other routine tasks. Without sufficient resources and support, this can affect GP wellbeing and potential burnout.

Step 1: Locating Existing Theories

We have developed an initial programme theory to describe our assumptions about important influences on GP workforce sustainability. This outlines potentially important contexts, active mechanisms and outcomes relating to GP workforce sustainability that we need to consider or build upon over the course of the review. Important contexts, for

For peer review only

example, might include organisation size, employment type, practice skill-mix, and nature of patient contact. Mechanisms could comprise continuity, peer dialogue, or clinical autonomy. A draft of our initial programme theory is provided in Figure 1.

**Figure 1** Initial programme theory – used at the beginning of realist review projects to map initial explanatory theories.

****Insert Figure 1 Around Here****

**Engaging PPI and stakeholder perspectives in expanding the initial programme theory: Exploring how to create joy**

In order to expand our initial programme theory and shape initial searches, we carefully planned a series of individual and small group discussions with PPI and stakeholder members to open a conversation about 'what creates joy in general practice and enables (or produces conditions for) 'equitable and effective patient care'. Participants were invited to draw on their own experiences and perspectives to explore this topic. Following introductions, we asked our PPI and stakeholder members to draw, describe, or write words which represented their thoughts, feelings or experiences about creating joy in general practice and enabling effective and equitable patient care. These were probed and discussed to build up a map of issues, including system level factors (e.g. patient access, nature of consultation interactions, interface between team members and other organisations, explicit patient facing work, and other 'hidden' work such as administration tasks). This map helped to modify and expand our initial programme theory, which was then used to shape initial searches and became the starting point for our review. An overview of the key concepts from our PPI and stakeholder

For peer review only

discussions can be found in our word cloud (Figure 2). A draft of our expanded initial programme theory is provided in Figure 3.

**Figure 2:** Word cloud visualisation

****Insert Figure 2 Around Here****

**Figure 3:** Expanded initial programme theory after stakeholder and PPI consultation workshops.

****Insert Figure 3 Around Here****

Step 2: Searching for Evidence

We will conduct searches to assemble a relevant body of literature that contains data we can use to develop and refine the initial programme theory developed in Step 1. To help us work efficiently, we will re-use search strategies employed in our scoping review and extend and update these to help us to identify new, relevant material also indexed in additional databases (CINAHL, HMIC, Web of Science - SCI-EXPANDED and SSCI indexes). These searches combined free text and subject heading terms for GPs with terms related to workforce and retention outcomes. We will conduct searches from other data sources including relevant organisational websites (e.g., Health Foundation, King's Fund, RCGP, DHSC) to identify grey literature.

We will limit our searches to identify material published in the English language. No limits will be placed on study design, as all relevant evidence (including opinion and

For peer review only

commentary) can be used to inform programme theory development. As the review progresses, we will identify additional material via citation searching, personal contacts and alerting services. Our initial inclusion criteria will include GP as the object of discussion or participant, discussion or study of UK general practice setting (including out-of-hours), include enquiry or discussion of GP workforce and/or GP work (e.g. nature, role, change, shift), relationship between GP and work or meaning attributed to work, and documents published within the last ten years.

Throughout the review, discussions within the project team and with PPI and stakeholders will be on-going, framing the focus and direction of literature selection, extraction, and analysis, and informing the production of the final programme theory configurations. At each stage, the team will ask 'is this relevant to the programme theory?', focusing on the connection between 'GP workforce sustainability' and 'effective and equitable patient care' to inform decisions about whether to explore and include certain areas of evidence and literature.

If necessary, we will conduct additional searches later in the project, with the aim of identifying data to fill gaps or refine specific aspects of our developing programme theory. Our additional searches will be guided by the input of our PPI and stakeholder groups, as we work with them to prioritise areas of the developing programme theory for exploration. Crucially, we will explore key workforce sustainability concepts (see above) with our PPI and stakeholders, alongside the UN definition of sustainable practice which includes working environment, resources, and people interacting within that system (14).

Step 3: Article Selection

For peer review only

We will screen documents for inclusion in the review in a three-step process: screening by title/abstract, following by screening in full text, with final selection resting on an assessment of each document’s relevance (whether it contains data relating to relevant contexts, mechanisms, outcomes or the relationships between these) and rigour (whether the methods used to generate each piece of data are credible and trustworthy) (21). To ensure consistency in this process, a 10% sample of decisions made at each stage will be independently checked by research team members (including CD, ER, BB and LL). Any discrepancies or disagreements will be discussed with ECO and the research team, and an agreed resolution documented.

Step 4: Data Extraction

Included full text documents will be uploaded to NVivo (qualitative data analysis software) for coding. Coding will be inductive (codes created to categorise data as reported in the included documents), deductive (some codes will be created in advance, based on the initial programme theory in Step 1) and retroductive (codes created based on an interpretation of the data, to infer what hidden causal forces may generate outcomes). Each new piece of relevant data identified will be used to refine and develop our programme theory and as the review progresses, included documents will be re-scrutinised to identify relevant data that may have been missed earlier, or as the programme theory developed. As with our screening and selection processes, a 10% sample of included documents will be independently checked by CD, ER, BB and LL to ensure consistency. Any discrepancies or disagreements will be discussed within the research team and documented.

Step 5: Synthesising the Evidence and Drawing Conclusions

For peer review only

We will use a realist logic of analysis to make sense of the extracted data and develop CMOCs that offer explanations for outcomes relating to GP workforce sustainability and provision of effective, equitable patient care. We will use interpretive cross-case comparison to understand and explain how and why observed outcomes occur. For example, we will compare documents that describe contexts in which are reported to enable GPs to flourish and sustain their working roles, to understand how contexts influence GP workforce sustainability and support effective and equitable patient care.

We will use a proven analysis and synthesis process (36). In brief, to operationalise our realist analysis, we will ask the following questions about the included documents and data therein:

- Interpretation of meaning: do the documents provide data that may be interpreted as functioning as context(s), mechanism(s), or outcome(s)?
- Interpretations and judgements about CMOCs: what is the CMOC for the data that has been interpreted as functioning as context, mechanism, or outcome?
- Interpretations and judgements about programme theory: how does this CMOC relate to the initial programme theory?

At the end of the process, we will produce a refined programme theory, underpinned by the data extracted from included documents, on which our co-production activities will build for dissemination of review findings.

Ethics and Dissemination

Dissemination

For peer review only

Our PPI co-applicants and stakeholders will help us to decide on the content, storyboarding and format (e.g., websites, leaflets, videos, social media). In addition to the final report, we will produce:

1. **Academic outputs**: e.g., protocol publication. Findings (to be submitted to a high-impact, open-access, and peer-reviewed journal), as well as tailored papers to different disciplinary journals.

2. **Audience-specific practitioner** 'how to' publications which outline practical advice on ways to maximise the sustainability of the GP workforce to support effective and equitable patient care.

3. **User-friendly summaries** of the review findings tailored to the needs of different audiences including the public and service users.

Our dissemination strategy will build upon a participatory approach, embedding PPI co-applicants and stakeholder involvement throughout the development of this research and project timeline, including opportunities for co-authorship and co-presenting. On-going engagement with key stakeholders will maximise opportunities to utilise our established networks, communication channels and links to policymakers and providers. Our approach will be integrative, valuing the different forms of knowledge needed to produce findings capable of informing complex decision-making within policy and practice.

Our audiences are:

1. Policymakers, decision-makers, and commissioners who will be key to implementing recommendations from our review.
2. Providers and practitioners whom we will brief on our findings.

For peer review only

3. Members of the public and charities - for whom we will tailor press releases, social media posts and engage directly via our PPI and stakeholder networks. Our migrant PPI members will help devise ways to best disseminate our outputs to non-English speaking communities.

## Ethics

Ethical approval is not required for this review as only secondary data sources will be used.

## DISCUSSION

## Importance of the Research

Our research will generate new knowledge about the interdependencies between contextual factors, causal mechanisms, and outcomes of interest. The findings and refined programme theory may be used to inform strategies and interventions intended to support, facilitate, and assist the GP workforce in delivering equitable and effective patient care. We will identify critical gaps in knowledge and prioritise the expectations for scope and nature of future GP work.

## Authors' contributions

The realist review was conceptualised by SP. SP wrote the first draft of this protocol and it was reviewed and revised by ECO, CD, TR, CG, TC, CH, MO, ELR, BB, EIL, and GV. The search strategy was developed by CD in consultation with ECO and the wider project team. All authors read and approved the final manuscript. SP is the guarantor.

## Funding Acknowledgement

For peer review only

This project is funded by the National Institute for Health Research (NIHR) School for Primary Care Research (SPCR593). Tim Rapley is a deputy theme lead within the NIHR Applied Research Collaboration Northeast and North Cumbria (NIHR200173). The views expressed are those of the authors and not necessarily those of the NIHR or the Department of Health and Social Care.

Competing interests

The authors have no conflicts of interests to declare.

## References

1. Owen K, Hopkins T, Shortland T, Dale J. GP retention in the UK: a worsening crisis. Findings from a cross-sectional survey. BMJ Open. 2019;9(2):e026048.
2. Iacobucci G. Burnout is harming GPs' health and patient care, doctors warn. British Medical Journal Publishing Group; 2021.
3. NHS E. The NHS Long Term Plan 2019 [Available from: https://www.longtermplan.nhs.uk/wp-content/uploads/2019/08/nhs-long-term-plan-version-1.2.pdf.
4. Hodes S, Hussain S, Jha N, Toberty L, Welch E. If general practice fails, The NHS fails: thebmjopinion 2021 [Available from: https://blogs.bmj.com/bmj/2021/05/14/if-general-practice-fails-the-nhs-fails/.
5. Hann M, Reeves D, Sibbald B. Relationships between job satisfaction, intentions to leave family practice and actually leaving among family physicians in England. The European Journal of Public Health. 2011;21(4):499-503.
6. Long L, Moore D, Robinson S, Sansom A, Aylward A, Fletcher E, et al. Understanding why primary care doctors leave direct patient care: a systematic review of qualitative research. BMJ Open. 2020;10(5):e029846.
7. Mol A. The logic of care: Health and the problem of patient choice: Routledge; 2008.
8. BMA. Pressures in general practice data analysis 2023 [Available from: https://www.bma.org.uk/advice-and-support/nhs-delivery-and-workforce/pressures/pressures-in-general-practice-data-analysis.
9. Beech J, Fraser C, Gardner T, Buzelli L, Williamson S, Alderwick H. Stressed and overworked: what the Commonwealth Fund's 2022 International Health Policy Survey of Primary Care Physicians in 10 Countries means for the UK. Health Foundation. Mar 2023.
10. Hobbs FDR, Bankhead C, Mukhtar T, Stevens S, Perera-Salazar R, Holt T, et al. Clinical workload in UK primary care: a retrospective analysis of 100 million consultations in England, 2007-14. Lancet. 2016;387(10035):2323-30.
11. Sinnott C, Moxey JM, Marjanovic S, Leach B, Hocking L, Ball S, et al. Identifying how GPs spend their time and the obstacles they face: a mixed-methods study. British Journal of General Practice. 2022;72(715):e148-e60.
12. Khan N. 'Hidden'no more: the real work of the 'part-time'GPs working full-time hours. British Journal of General Practice. 2023;73(726):26-7.

For peer review only

13. Freeman G, Hughes, J. Continuity of care and the patient experience 2010 [Available from: https://www.kingsfund.org.uk/sites/default/files/field/field_document/continuity-care-patient-experience-gp-inquiry-research-paper-mar11.pdf.
14. Sandvik H, Hetlevik, O, Blinkenberg, J, Hunskaar, S. Continuity in general practice as predictor of mortality, acute hospitalisation, and use of out-of-hours care: a registry-based observational study in Norway. British Journal of General Practice. 2022;72(715).
15. Kajaria-Montag H, Freeman M, Scholtes S. Continuity of care increases clinical productivity in primary care. 2021.
16. Laurant M, Harmsen M, Wollersheim H, Grol R, Faber M, Sibbald B. The impact of nonphysician clinicians. Medical Care Research and Review. 2009;66(6_suppl):36S-89S.
17. Cadogan SL, Browne JP, Bradley CP, Fitzgerald AP, Cahill MR. Physician and practice characteristics associated with immunoglobulin test ordering. Family Practice. 2018;35(1):41-6.
18. Morgan S, Henderson KM, Tapley A, Scott J, Van Driel ML, Spike NA, et al. Pathology test-ordering behaviour of Australian general practice trainees: a cross-sectional analysis. International Journal for Quality in Health Care. 2015;27(6):528-35.
19. Grigoroglou C, Walshe K, Kontopantelis E, Ferguson J, Stringer G, Ashcroft DM, et al. Locum doctor use in English general practice: analysis of routinely collected workforce data 2017–2020. British journal of general practice. 2022;72(715):e108-e17.
20. Nussbaum C, Massou E, Fisher R, Morciano M, Harmer R, Ford J. Inequalities in the distribution of the general practice workforce in England: a practice-level longitudinal analysis. BJGP Open. 2021;5(5).
21. Pawson R. Evidence-Based Policy: A Realist Perspective London: SAGE Publications.; 2006.
22. Marchand C, Peckham S. Addressing the crisis of GP recruitment and retention: a systematic review. Br J Gen Pract. 2017;67(657):e227-e37.
23. Andah E, Essang B, Friend C, Greenley S, Harvey K, Spears M, et al. Understanding the impact of professional motivation on the workforce crisis in medicine: a rapid review. BJGP Open. 2021;5(2).
24. Verma P, Ford JA, Stuart A, Howe A, Everington S, Steel N. A systematic review of strategies to recruit and retain primary care doctors. BMC Health Serv Res. 2016;16:126.
25. Lachish S, Svirko E, Goldacre MJ, Lambert T. Factors associated with less-than-full-time working in medical practice: results of surveys of five cohorts of UK doctors, 10 years after graduation. Human resources for health. 2016;14:1-11.
26. Fletcher E, Abel GA, Anderson R, Richards SH, Salisbury C, Dean SG, et al. Quitting patient care and career break intentions among general practitioners in South West England: findings of a census survey of general practitioners. BMJ open. 2017;7(4):e015853.
27. Dale J, Potter R, Owen K, Leach J. The general practitioner workforce crisis in England: a qualitative study of how appraisal and revalidation are contributing to intentions to leave practice. BMC family practice. 2016;17:1-7.
28. Doran N, Fox F, Rodham K, Taylor G, Harris M. Lost to the NHS: a mixed methods study of why GPs leave practice early in England. British journal of general practice. 2016;66(643):e128-e35.
29. Smith F, Goldacre MJ, Lambert TW. Retirement ages of senior UK doctors: national surveys of the medical graduates of 1974 and 1977. BMJ open. 2018;8(6):e022475.
30. Campbell JL, Fletcher E, Abel G, Anderson R, Chilvers R, Dean SG, et al. Policies and strategies to retain and support the return of experienced GPs in direct patient care: the ReGROUP mixed-methods study. 2019;7:1-288.
31. Park S, Berlin A. 'Divided we fail': understanding inter-relations and the role of the generalist. British Journal of General Practice. 2012;62(604):596-8.
32. Bansal A, Greenley S, Mitchell C, Park S, Shearn K, Reeve J. Optimising planned medical education strategies to develop learners' person-centredness: A realist review. Medical Education. 2022;56(5):489-503.
33. Pereira Gray DJ, Sidaway-Lee K, White E, Thorne A, Evans PH. Continuity of care with doctors-a matter of life and death? A systematic review of continuity of care and mortality. BMJ Open. 2018;8(6):e021161.

For peer review only

489 34. Abrams R, Wong G, Mahtani KR, Tierney S, Boylan A-M, Roberts N, et al. Delegating home visits in general practice: a realist review on the impact on GP workload and patient care. British Journal of General Practice. 2020;70(695):e412-e20.
492 35. Bienkowska-Gibbs T, King S, Saunders C, Henham M. New organisational models of primary care to meet the future needs of the NHS: a brief overview of recent reports. RAND EUROPE 2015 2015.
495 36. Papoutsi C, Mattick K, Pearson M, Brennan N, Briscoe S, Wong G. Interventions to improve antimicrobial prescribing of doctors in training (IMPACT): a realist review. 2021.

For peer review only

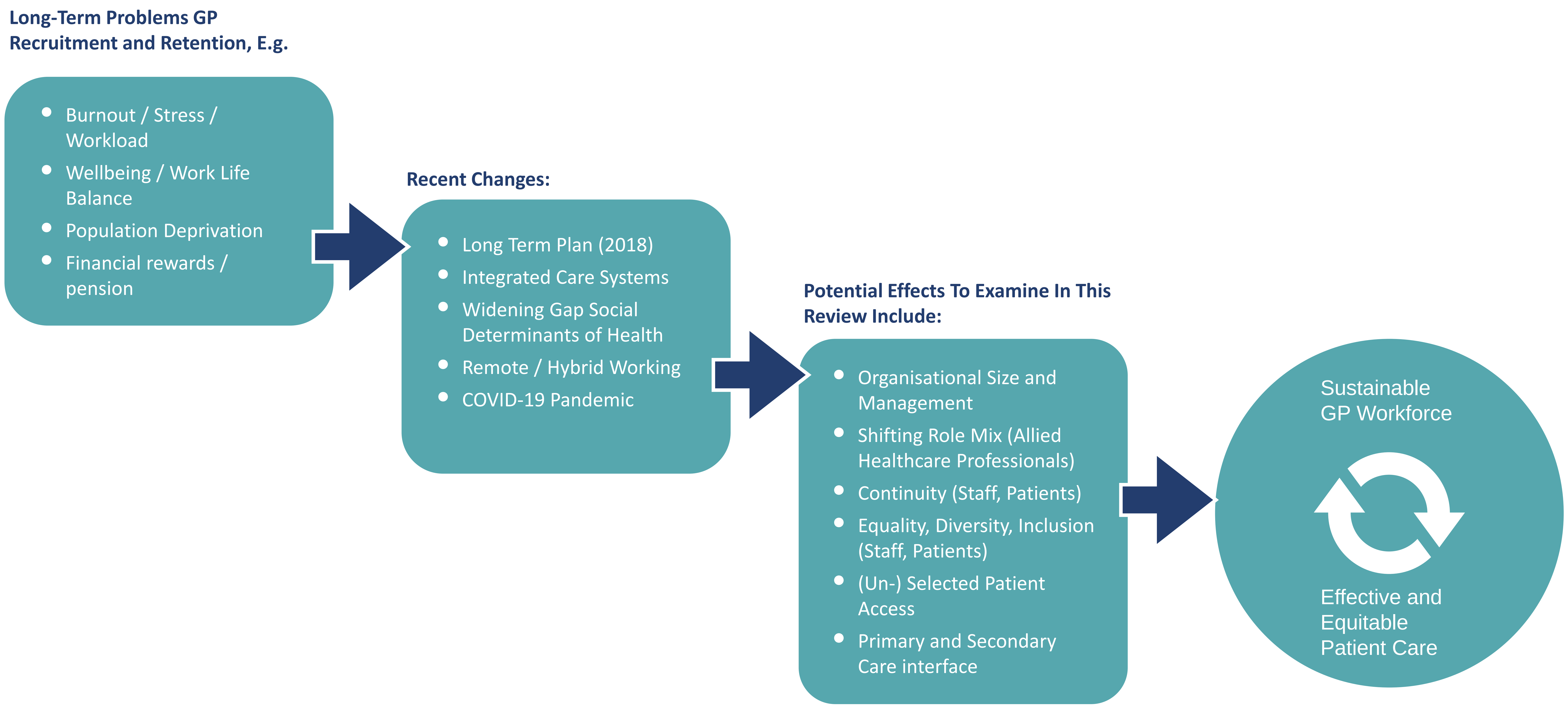
Long-Term Problems GP
Recruitment and Retention, E.g.
Burnout / Stress / Workload
Wellbeing / Work Life Balance
Population Deprivation
Financial rewards / pension
Recent Changes:
Long Term Plan (2018)
Integrated Care Systems
Widening Gap Social Determinants of Health
Remote / Hybrid Working
COVID-19 Pandemic
Potential Effects To Examine In This Review Include:
Organisational Size and Management
Shifting Role Mix (Allied Healthcare Professionals)
Continuity (Staff, Patients)
Equality, Diversity, Inclusion (Staff, Patients)
(Un-) Selected Patient Access
Primary and Secondary Care interface
Sustainable GP Workforce
Effective and Equitable Patient Care

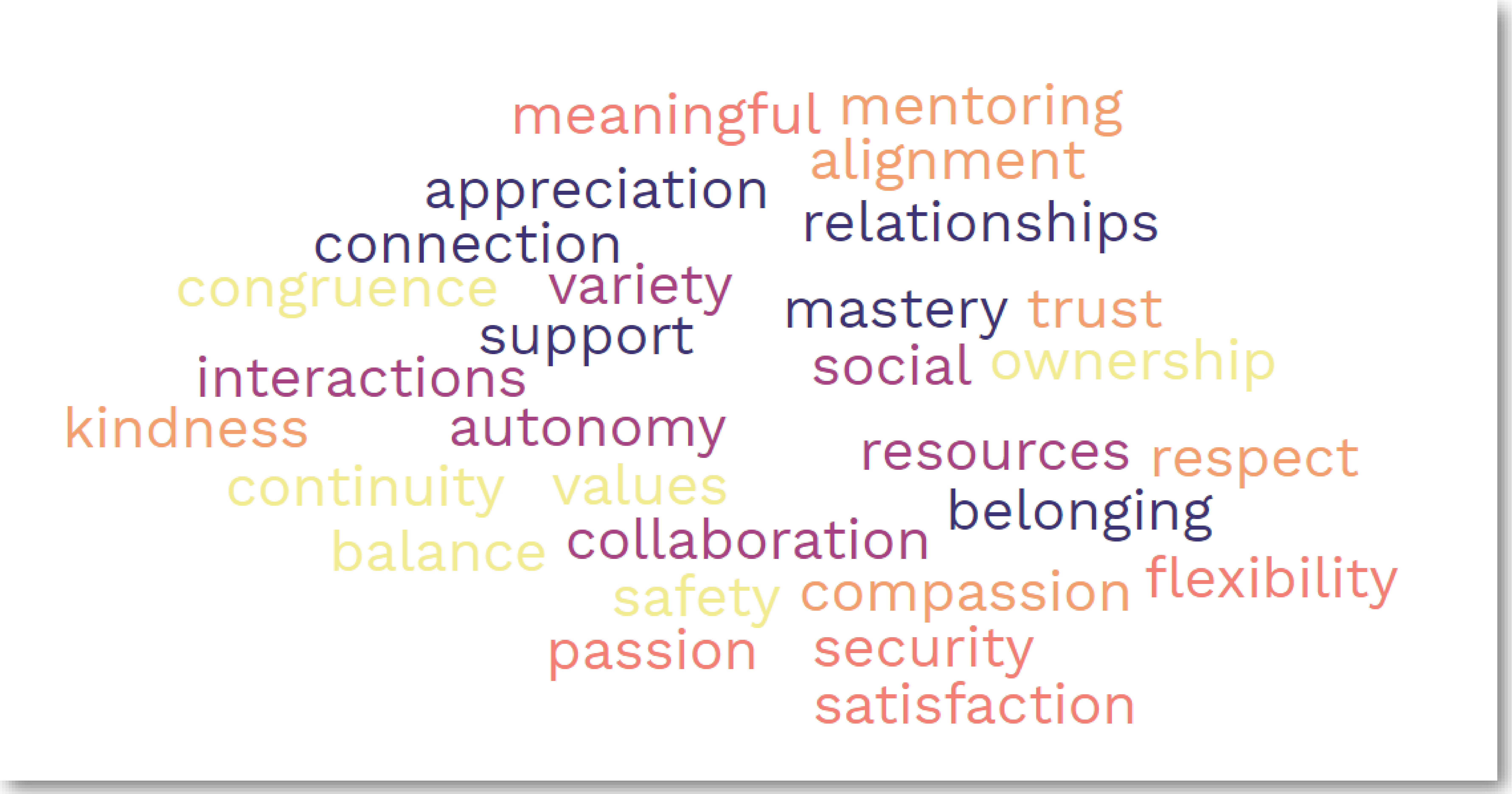
meaningful
mentoring
alignment
appreciation
relationships
connection
congruence
variety
mastery
trust
support
social
ownership
interactions
kindness
autonomy
resources
respect
continuity
values
belonging
balance
collaboration
flexibility
safety
compassion
passion
security
satisfaction

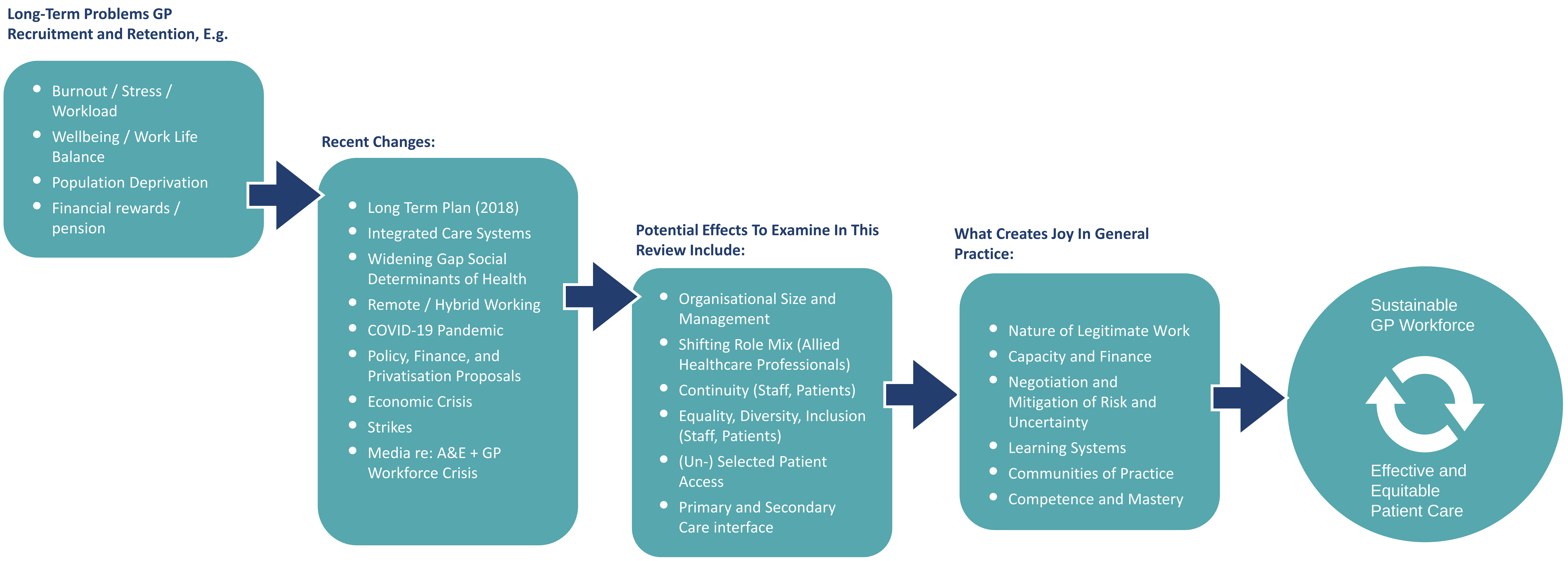
Long-Term Problems GP Recruitment and Retention, E.g.
Burnout / Stress / Workload
Wellbeing / Work Life Balance
Population Deprivation
Financial rewards / pension
Recent Changes:
Long Term Plan (2018)
Integrated Care Systems
Widening Gap Social Determinants of Health
Remote / Hybrid Working
COVID-19 Pandemic
Policy, Finance, and Privatisation Proposals
Economic Crisis
Strikes
Media re: A&E + GP Workforce Crisis
Potential Effects To Examine In This Review Include:
Organisational Size and Management
Shifting Role Mix (Allied Healthcare Professionals)
Continuity (Staff, Patients)
Equality, Diversity, Inclusion (Staff, Patients)
(Un-) Selected Patient Access
Primary and Secondary Care interface
What Creates Joy In General Practice:
Nature of Legitimate Work
Capacity and Finance
Negotiation and Mitigation of Risk and Uncertainty
Learning Systems
Communities of Practice
Competence and Mastery
Sustainable GP Workforce
Effective and Equitable Patient Care

BMJ Open

# General practitioner workforce sustainability to maximise effective and equitable patient care: a realist review protocol.

| | |
|---|---|
| Journal: | *BMJ Open* |
| Manuscript ID | bmjopen-2023-075189.R1 |
| Article Type: | Protocol |
| Date Submitted by the Author: | 22-Jan-2024 |
| Complete List of Authors: | Park, Sophie; University College London, Department of Primary Care and Population Health<br>Owen, Emily; University College London, Department of Primary Care & Population Health<br>Burford, Bryan; Newcastle University, School of Medicine<br>Cohen, Tanya; University College London, Department of Primary Care and Population Health<br>Duddy, Claire; University of Oxford, Nuffield Department of Primary Care Health Sciences<br>Dunn, Harry; University of Cambridge, School of Clinical Medicine<br>Fadia , Vacha ; University College London, Department of Primary Care & Population Health<br>Goodman, Claire; University of Hertfordshire, Centre for Research in Public Health and Community Care<br>Henry , Cecily ; University College London, Department of Primary Care and Population Health<br>Lamb , Elizabeth; Newcastle University, School of Medicine<br>Ogden , Margaret; University College London, Department of Primary Care and Population Health<br>Rapley, Tim; Northumbria University, Department of Social Work, Education and Community Wellbeing<br>Rees, Eliot; University College London, Department of Primary Care and Population Health; Keele University, School of Medicine<br>Vance, Gillian ; Newcastle University, School of Medicine<br>Wong, Geoff; University of Oxford, Nuffield Department of Primary Care Health Sciences |
| <b>Primary Subject Heading</b>: | General practice / Family practice |
| Secondary Subject Heading: | Health services research, Sociology, Public health, Health policy |
| Keywords: | Health Equity, Organisation of health services < HEALTH SERVICES ADMINISTRATION & MANAGEMENT, Patient-Centered Care, Physicians, Primary Care < Primary Health Care |
| | |

SCHOLARONE™
Manuscripts

RUNNING HEAD: REALIST REVIEW PROTOCOL

# General practitioner workforce sustainability to maximise effective and equitable patient care: a realist review protocol.

Sophie Park[1], Emily C. Owen[1], Bryan Burford[2], Tanya Cohen[1], Claire Duddy[3], Harry Dunn[4], Vacha Fadia[1], Claire Goodman[5], Cecily Henry[1], Elizabeth I. Lamb[2], Margaret Ogden[1], Tim Rapley[6], Eliot L. Rees[1,7], Gillian Vance[2], Geoff Wong[3]

[1]Department of Primary Care and Population Health, University College London, London, UK, [2] School of Medicine, Newcastle University, Newcastle Upon Tyne, UK, [3]Nuffield Department of Primary Care Health Sciences, University of Oxford, Oxford, UK, [4]School of Clinical Medicine, University of Cambridge, Cambridge, UK, [5]Centre for Research in Public Health and Community Care, University of Hertfordshire, Hatfield, UK, [6]Department of Social Work, Education and Community Wellbeing, Northumbria University, Newcastle Upon Tyne, UK, [7]School of Medicine, Keele University, Keele, UK.

Corresponding author:

Dr Emily C. Owen; UCL Research Department of Primary Care & Population Health; Royal Free Campus; Hampstead, London; NW3 2PY.

Email: Emily.owen@ucl.ac.uk; Phone: 020 7794 0500.

Keywords: General Practice; Workforce; Sustainability; Healthcare system; Retention.

Word count: 5699.

For peer review only

ABSTRACT

**Introduction:** There are not enough General Practitioners (GPs) in the UK National Health Service. This problem is worse in areas of the country where poverty and underinvestment in health and social care mean patients experience poorer health compared with wealthier regions. Encouraging more doctors to choose and continue in a GP career is a government priority. This review will examine which aspects of the healthcare system affect GP workforce sustainability, how, why, and for whom.

**Methods and analysis:** A realist review is a theory-driven interpretive approach to evidence synthesis, that brings together qualitative, quantitative, mixed-methods research, and grey literature. We will use a realist approach to synthesise data from the available published literature to refine an evidence-based programme theory that will identify the important contextual factors and underlying mechanisms that underpin observed outcomes relating to GP workforce sustainability. Our review will follow Pawson's five iterative stages: (1) finding existing theories; (2) searching for evidence; (3) article selection; (4) data extraction; and (5) synthesising evidence and drawing conclusions. We will work closely with key stakeholders and embed patient and public involvement throughout the review process to refine the focus of the review and enhance the impact and relevance of our research.

**Ethics and dissemination:** This review does not require formal ethical approval as it draws on secondary data from published articles and grey literature. Findings will be disseminated through multiple channels, including publication in peer-reviewed journals, at national and international conferences, and other digital scholarly communication tools such as video summaries, X, and blog posts.

**PROSPERO registration number**: CRD42023395583.

For peer review only

Strengths and limitations of this study:

- This is the first systematic review to use realist methodology to explore which aspects of the healthcare system affect GP workforce sustainability, how, why, and for whom.
- We will include a diverse range of evidence, including, empirical articles, conference materials, policy reports, and editorials, increasing review depth and breadth.
- We will embed patient and public involvement and stakeholders throughout the design, analysis, and report stages of the project, enhancing impact and relevance.
- We will only include documents that are written or translated in English.
- Our context-mechanism-outcome configurations and refined programme theory may be limited by the availability, quality, and richness of existing identified literature in this field.

For peer review only

# INTRODUCTION

UK general practice has been described as being in crisis (1). A shortage and exodus (2) of General Practitioners (GPs) is an urgent and challenging problem, attracting significant media attention and widespread public debate. The National Health Service (NHS) Long Term Plan proposed a net increase of 5000 GPs, alongside expectations of work distribution and re-allocation across primary care teams (3). The impact of these changes on the GP workforce remains relatively unexplored. In the UK, general practice provides comprehensive (a patient may present with any type of problem), person-centred (holistic caring and individualised care), and universal (everyone can attend) access to healthcare. The ways in which this care is organised, and delivered do tend, therefore, to differ depending on the needs of local communities, available resources, and capacity (4).

It is widely acknowledged that general practice provides 90% of patient care utilising approximately 8% of the NHS budget (5), meaning this is a key element of broader debates about a whole-system NHS moral and financial crisis. Existing research has focused on 'numbers in' 'numbers out' calculations of workforce need, predictions, and requirements. GPs' decisions to stay or leave are often positioned as an individual 'choice' (6, 7, 8), resilience, or failure, with less attention given to the social and organisational milieu in which those decisions are produced. A systems approach recognises the need to acknowledge this inherent dynamic complexity, by studying the interconnected components (e.g. the nature of social interactions between patients, colleagues, and external institutions, alignment of personal and policy priorities, and organisational support structures) that work together in an integrated and coordinated way to sustain and enhance the GP workforce.

The figures reflecting shifts in the number of GPs available are well-rehearsed. For example, as of February 2023, the number of full-time equivalent GPs has fallen significantly

For peer review only

(approximately 2,100 fewer fully qualified full-time GPs compared to September 2015) (9), many GPs are approaching traditional retirement ages, and the challenges of recruiting and retaining GPs in rural and coastal areas is well recognised, both nationally and internationally (10). UK GPs have reported higher levels of emotional distress than GPs in nearly all other countries (11). In parallel, readily measurable elements of GP work have been reported to increase, with 37.2 million consultations estimated to have been delivered by practices and primary care networks in October 2023 (12). Consultations are reported to have increased in length (13), alongside changes in form (e.g. use of technology). Measures of GP time spent on operational challenges and problems are high (14) and the 'hidden', non-patient-facing work of GPs is increasingly being recognised (15). It is widely acknowledged that GPs staying in practice has benefits for both patient care (16, 17), and practice productivity (18). For example, experienced GPs work more efficiently, lessening the scale of patient medicalisation, investigation, and treatment, and requiring shorter appointment times compared to trainees and other healthcare professionals (19, 20, 21). We can also see that practices with a higher proportion of patients in deprived areas; lower care quality commission ratings; single-handed and rural practices, have fewer GPs overall and more locums (22) emphasising the importance of wider contextual factors in shaping the nature of work and workforce patterns (23).

A systems approach is well suited as a basis for examining the multitude of factors influencing the GP workforce and how these ultimately shape career decisions. By taking a system and theory-driven realist approach to understanding what works, in which contextual circumstances, and to what extent, we aim to make visible the often unsaid or implicit issues that influence working environments, relationships, and organisational culture. The findings from our review will offer structural and organisational recommendations to inform sustainable, context-specific ways of future working, which can underpin effective and

equitable patient care. For the purposes of our review, we define equitable patient care as the process through which healthcare systems maximise inclusive ways to facilitate access and care delivery for patients. While equality implies delivery of the same for all, equity focuses on ensuring patients have the best opportunity to reach needed outcomes, even if systems must change or adapt processes, to enable this. Effective patient care may vary according to the patient's needs and contextual circumstances but will include the best possible fit for them at that particular point in time (4).

METHODS

**Review aim, research objectives, and research questions**

**Aim**

This review aims to examine which aspects of the healthcare system affect GP workforce sustainability, how, why, and for whom.

**Research Objectives:**

1. Develop a programme theory through an evidence synthesis of how the GP workforce can be sustainable and work effectively with others to support effective and equitable patient care.

2. Embed and respond to patient and public involvement (PPI) and stakeholder perspectives throughout the design, analysis, and report stages of the project, thus maximising the relevance and utility of review findings.

For peer review only

3. Make recommendations for practice and policy based on the refined programme theory.

**Research Questions:**

What can we learn from the existing literature that will promote GP workforce sustainability, to support effective and equitable patient care? Specifically:

1. Which mechanisms produce intended and/or unintended outcomes in GP workforce sustainability?

2. Which contexts influence whether different mechanisms produce intended and/or unintended outcomes in GP workforce sustainability?

3. Which interventional strategies lead to intended and/or unintended outcomes for GP workforce sustainability?

Approach

We will conduct a realist review, synthesising data from the published literature to produce a refined evidence-based programme theory. This will identify the important contexts and mechanisms which underpin observed outcomes relating to GP workforce sustainability to support the delivery of effective and equitable patient care. A realist review is an interpretive and theory-driven approach to evidence synthesis, that brings together data drawn from quantitative, qualitative, and mixed methods research, as well as the wider grey literature (24). Using this data and a realist logic of analysis will allow us to examine diverse evidence with a clear focus on understanding factors which support or challenge GP workforce sustainability, how these are shaped by important contexts, and the mechanisms that underpin them. Based on this understanding, captured in our final programme theory, we will be able

For peer review only

to identify and prioritise important system-level contexts that may be amenable to change. Our review began in January 2023 and will end December 2025.

Initial scoping searches using EMBASE, MEDLINE and PsycINFO identified a range of qualitative and quantitative primary and secondary evidence focused on GP retention. We have identified four existing reviews focused on GP retention (7, 25, 26, 27), and four additional studies from broader scoping searches, including one examining factors associated with part-time working (28). Existing studies on this topic are often descriptive, identifying a complex array of factors associated with workforce attrition without theoretical analysis. These include individual issues (e.g. wellbeing, burnout, identity), practice (e.g. workload, administrative burden, clinical autonomy (25) - and opportunities for continuity of care (7, 26), and national (e.g. revalidation) contexts (1, 29, 30, 31). One study found the intrinsic attractions of retirement (e.g. increased time for leisure') contributed to GPs leaving practice (32). Factors shaping GP retention in one UK region (33) were described as 'push' (workload, stress, job dissatisfaction, and organisational injustice de-motivating factors) and 'pull' (work-life balance).

This review will attend to the complex systems in which GPs work, in order to better understand the factors which, keep, sustain, and enable GPs to flourish within their work. A realist approach will allow us to examine a wide range of literature about the system and related social interactions (e.g. across the primary and secondary care interface, between patients and clinicians), and to integrate patient and stakeholder experience to inform our approach and produce new ways of thinking about this challenging field. We will identify causal explanations to illuminate the connections between GP work (e.g. nature and scope) and GP career intentions. Depending on the available evidence, we aim to examine a range of factors and circumstances (e.g. location, employment arrangements such as small or large

For peer review only

institutions, salaried, partnered, payment by performance), systems of support inside or outside practices, and patient deprivation. We will examine the relationships between these contextual factors and a range of reported outcomes. This will help to identify the circumstances in which a sustainable and flourishing GP workforce for delivery of effective and equitable patient care can be realised.

Our approach will follow Pawson's (24) five iterative stages for realist reviews detailed below. This begins with an initial programme theory developed through scoping searches and preliminary consultation with stakeholders and patients. As the review progresses, we will gather and interpret data from the literature to refine and develop our explanations of important outcomes, drawing on the expertise of our PPI and stakeholder groups. At the end of the project, our final programme theory will consist of evidenced context-mechanism-outcome configurations (CMOCs), presenting realist causal explanations for outcomes relating to the GP workforce sustainability to support effective and equitable patient care.

**Patient and Public Involvement**

We have three PPI co-applicants within our core research team. The team also includes professionals from a range of disciplinary backgrounds including general practice, secondary care, nursing, psychology, and sociology. Additionally, we have recruited six PPI contributors from coastal, rural, and diverse urban contexts. An equality of opportunity approach will be taken throughout this process, enabling us to respond in an agile way to maximise PPI involvement. For example, helping to refine the programme theory, shape our findings and contribute to both the format and content of our outputs (e.g., written word, infographics), and ensuring that proposed solutions are both feasible and acceptable. Finally, our PPI members will play an essential role in developing our dissemination strategy, and the format and content of the outputs of this project. We also have a stakeholder group including

For peer review only

professionals representing a variety of GP employment models, paramedic, pharmacy, policy, practice, management, and educational backgrounds.

In the development of this proposal, we invited PPI and stakeholders to share their priorities. GPs often work at the interface between human experience and the technicalities of pathology, negotiating boundaries and overlaps between stress, illness and disease (34). Person-centred care has been recognised as a key element of this process (35, 36). Knowledge of the 'person' can be established over time, building on trust and relational currency between patient and practitioner (37). One key area of concern in PPI and stakeholder discussions during development of this review protocol, was 'care of other'. GPs spoke about the importance of person-centred care, while PPI co-applicants highlighted the importance of looking after practitioners and supporting their well-being. As one PPI contributor explained, if patients are treated as 'a number' within the healthcare system, this is potentially mirrored in their treatment and expectation of healthcare professionals. One key area about GP workforce sustainability in this review, therefore, is the relational elements of GP work, and how these may support or undermine workforce sustainability.

We invited PPI and stakeholders to help produce three case study examples about GP workforce sustainability:

**Case Study 1.** In the UK, following the introduction of the NHS Long Term Plan, multi-disciplinary team working in general practice is increasing (3). Many practices now employ pharmacists, paramedics, physician associates, physiotherapists, and mental health professionals. In some circumstances, this has enabled GPs to focus on the clinical care of particularly 'complex' patients (e.g. with multimorbidity), delegate some home visits (38), and prescribing demands (39). Yet, some stakeholders report a) anecdotal increases in workload relating to supervision and support of new staff and b) increased levels of follow up

For peer review only

(e.g. lower thresholds for initiating patient investigations), c) patients needing to attend multiple appointments to resolve a problem(s) and d) challenges negotiating access for patients with unselected and comprehensive healthcare problems to see the most appropriate professional, at the most appropriate time. These fast-moving changes to the GP role and patient access to GP care, have therefore been experienced both as producing opportunities and challenges.

**Case Study 2.** Relationships between GPs and secondary care colleagues can influence patient care and access to services. For example, if close and rapid communication is established (e.g. informal sharing of numbers, or formal routes for email advice and guidance), GPs can progress patient care, minimising referrals and waiting list delays, and maximising timely patient care delivery in the primary care setting. This could have an impact on a GP's sense of agency or autonomy to provide immediate care for patients, but also a sense of working as part of a distributed team, rather than alone. As an example: a specialist nurse managing a Paediatric Allergy clinic advice service. In one area, this enables access to advice regarding initial investigations and treatment. In another area, this increases recommendations to refer. This means waiting lists could be shortened or lengthened, expediting, or delaying assessment of urgent or severe patients, or periods of time requiring additional GP support.

**Case Study 3.** GPs sometimes develop 'special interests' (e.g. supporting patients with mental health problems). This can be positive for practices, patients, and GP professional development. Yet, it can also produce challenges and inequalities if not managed well. For example, patients requiring more clinical input, longer consultations and emotional investment being directed to seek help from particular GPs. Without adequate planning, this can result in GPs who support these patients running late, working longer hours (for no extra

For peer review only

pay) to complete other routine tasks. Without sufficient resources and support, this can affect GP wellbeing and potential burnout.

Step 1: Locating Existing Theories

We have developed an initial programme theory to describe our assumptions about important influences on GP workforce sustainability. This outlines potentially important contexts, active mechanisms and outcomes relating to GP workforce sustainability that we need to consider or build upon over the course of the review. Important contexts, for example, might include organisation size, employment type, practice skill-mix, and nature of patient contact. Mechanisms could comprise continuity, peer dialogue, or clinical autonomy. A draft of our initial programme theory is provided in Figure 1.

**Figure 1** Initial programme theory – used at the beginning of realist review projects to map initial explanatory theories.

****Insert Figure 1 Around Here****

**Engaging PPI and stakeholder perspectives in expanding the initial programme theory: Exploring how to create joy.**

To expand our initial programme theory and shape initial searches, we carefully planned a series of individual and small group discussions with PPI and stakeholder members to open a conversation about 'what creates joy in general practice and enables (or produces conditions for) 'equitable and effective patient care'. Participants were invited to draw on their own experiences and perspectives to explore this topic. Following introductions, we asked our PPI and stakeholder members to draw, describe, or write words which represented their thoughts,

For peer review only

feelings or experiences about creating joy in general practice and enabling effective and equitable patient care. These were probed and discussed to build up a map of issues, including system level factors (e.g. patient access, nature of consultation interactions, interface between team members and other organisations, explicit patient facing work, and other 'hidden' work such as administration tasks). This map helped to modify and expand our initial programme theory, which was then used to shape initial searches and became the starting point for our review. An overview of the key concepts from our PPI and stakeholder discussions can be found in our word cloud (Figure 2). A draft of our expanded initial programme theory is provided in Figure 3.

**Figure 2:** Word cloud visualisation

****Insert Figure 2 Around Here****

**Figure 3:** Expanded initial programme theory after stakeholder and PPI consultation workshops.

****Insert Figure 3 Around Here****

Step 2: Searching for Evidence

We will conduct searches to assemble a relevant body of literature that contains data we can use to develop and refine the initial programme theory developed in Step 1. To help us work efficiently, we will re-use search strategies employed in our scoping review and extend and update these to identify new, relevant material also indexed in additional databases (CINAHL, HMIC, Web of Science - SCI-EXPANDED and SSCI indexes). The

For peer review only

main databases we will search include: MEDLINE; Embase; PsychINFO; CINAHL; HMIC; and Web of Science. These searches will combine free text and subject heading (MeSH) terms for GPs with a range of terms relating to workforce and retention outcomes. The full details of the search developed for MEDLINE are available in online supplementary file 1. We will conduct searches from other data sources including relevant organisational websites (e.g., Health Foundation, King's Fund, RCGP, DHSC) to identify grey literature.

We will limit our searches to identify material published in the English language. No limits will be placed on study design, as all relevant evidence (including opinion and commentary) can be used to inform programme theory development. As the review progresses, we will identify additional material via citation searching, our PPI and stakeholder members, and alerting services. Our initial inclusion criteria will include GP as the object of discussion or participant, discussion, or study of UK general practice setting (including out-of-hours), include enquiry or discussion of GP workforce and/or GP work (e.g. nature, role, change, shift), relationship between GP and work or meaning attributed to work, and documents published within the last ten years.

Throughout the review, discussions within the project team and with PPI and stakeholders will be on-going, framing the focus and direction of literature selection, extraction, and analysis, and informing the production of the final programme theory configurations. At each stage, the team will ask 'is this relevant to the programme theory?', focusing on the connection between 'GP workforce sustainability' and 'effective and equitable patient care' to inform decisions about whether to explore and include certain areas of evidence and literature.

If necessary, we will conduct additional searches later in the project, with the aim of identifying data to fill gaps or refine specific aspects of our developing programme theory.

Our additional searches will be guided by the input of our PPI and stakeholder groups, as we work with them to prioritise areas of the developing programme theory for exploration. Crucially, we will explore key workforce sustainability concepts (see above) with our PPI and stakeholders, alongside the United Nations definition of sustainable practice which includes working environment, resources, and people interacting within that system (14).

Step 3: Article Selection

We will screen documents for inclusion in the review in a three-step process: screening by title/abstract, following by screening in full text, with final selection resting on an assessment of each document's relevance (whether it contains data relating to relevant contexts, mechanisms, outcomes or the relationships between these) and rigour (whether the methods used to generate each piece of data are credible and trustworthy) (24). To ensure consistency in this process, a 10% sample of decisions made at each stage will be independently checked by research team members (including CD, ER, BB and LL). Any discrepancies or disagreements will be discussed with ECO and the research team, and an agreed resolution documented.

Step 4: Data Extraction

Included full text documents will be uploaded to NVivo (qualitative data analysis software) for coding. Coding will be inductive (codes created to categorise data as reported in the included documents), deductive (some codes will be created in advance, based on the initial programme theory in Step 1) and retroductive (codes created based on an interpretation of the data, to infer what hidden causal forces may generate outcomes). Each new piece of relevant data identified will be used to refine and develop our programme theory and as the review progresses, included documents will be re-scrutinised to identify relevant data that

For peer review only

may have been missed earlier, or as the programme theory developed. As with our screening and selection processes, a 10% sample of included documents will be independently checked by CD, ER, BB, and EL to ensure consistency. Any discrepancies or disagreements will be discussed within the research team and documented.

Step 5: Synthesising the Evidence and Drawing Conclusions

We will use a realist logic of analysis to make sense of the extracted data and develop CMOCs that offer explanations for outcomes relating to GP workforce sustainability and provision of effective, equitable patient care. We will use interpretive cross-case comparison to understand and explain how and why observed outcomes occur. For example, we will compare documents that describe contexts in which are reported to enable GPs to flourish and sustain their working roles, to understand how contexts influence GP workforce sustainability and support effective and equitable patient care.

We will use a proven analysis and synthesis process (40). In brief, to operationalise our realist analysis, we will ask the following questions about the included documents and data therein:

- Interpretation of meaning: do the documents provide data that may be interpreted as functioning as context(s), mechanism(s), or outcome(s)?
- Interpretations and judgements about CMOCs: what is the CMOC for the data that has been interpreted as functioning as context, mechanism, or outcome?
- Interpretations and judgements about programme theory: how does this CMOC relate to the initial programme theory?

For peer review only

At the end of the process, we will produce a refined programme theory, underpinned by the data extracted from included documents, on which our co-production activities will build for dissemination of review findings.

Ethics and Dissemination

Dissemination

Our PPI co-applicants and stakeholders will help us to decide on the content, storyboarding and format (e.g., websites, leaflets, videos, social media). In addition to the final report, we will produce:

1. **Academic outputs**: e.g., protocol publication. Findings (to be submitted to a high-impact, open-access, and peer-reviewed journal), as well as tailored papers to different disciplinary journals.
2. **Audience-specific practitioner** ‘how to’ publications which outline practical advice on ways to maximise the sustainability of the GP workforce to support effective and equitable patient care.
3. **User-friendly summaries** of the review findings tailored to the needs of different audiences including the public and service users.

Our dissemination strategy will build upon a participatory approach, embedding PPI co-applicants and stakeholder involvement throughout the development of this research and project timeline, including opportunities for co-authorship and co-presenting. On-going engagement with key stakeholders will maximise opportunities to utilise our established networks, communication channels and links to policymakers and providers. Our approach

For peer review only

will be integrative, valuing the different forms of knowledge needed to produce findings capable of informing complex decision-making within policy and practice.

Our audiences are:

1. Policymakers, health system decision-makers, and commissioners who will be key to implementing recommendations from our review.
2. Providers and practitioners whom we will brief on our findings.
3. Members of the public and charities - for whom we will tailor press releases, social media posts and engage directly via our PPI and stakeholder networks. Our migrant PPI members will help devise ways to best disseminate our outputs to non-English speaking communities.

Ethics

Ethical approval is not required for this review as only secondary data sources will be used.

## DISCUSSION

Importance of the Research

Following an escalation in changes affecting general practice during and after the COVID-19 pandemic, there are national and international debates about what general practice of the future should look like. We hope the findings from our review can influence political discussions and shape future patient care, contributing to the scholarship of the field, and to a more sustainable general practice. We will generate new knowledge about the interdependencies between contextual factors, causal mechanisms, and outcomes of interest. The findings may be used to inform strategies and interventions intended to support, facilitate, and assist the GP workforce in delivering equitable and effective patient care. We

For peer review only

will identify critical gaps in knowledge and prioritise the expectations for scope and nature of GP work.

Authors' contributions

The realist review was conceptualised by SP. SP wrote the first draft of this protocol and it was reviewed and revised by ECO, BB, TC, CD, HD, VF, CG, CH, EIL, MO, TR, ELR, GV, and GW. The search strategy was developed by CD in consultation with ECO and the wider project team. All authors read and approved the final manuscript. SP is the guarantor.

Funding Acknowledgement

This project is funded by the National Institute for Health Research (NIHR) School for Primary Care Research (SPCR593). Tim Rapley is a deputy theme lead within the NIHR Applied Research Collaboration Northeast and North Cumbria (NIHR200173). Emily Owen, Sophie Park, and Geoff Wong are members of the NIHR SPCR Evidence Synthesis Working Group. The views expressed are those of the authors and not necessarily those of the NIHR or the Department of Health and Social Care.

Competing interests

The authors have no conflicts of interests to declare.

## References

1. Owen K, Hopkins T, Shortland T, Dale J. GP retention in the UK: a worsening crisis. Findings from a cross-sectional survey. BMJ Open. 2019;9(2):e026048.
2. Iacobucci G. Burnout is harming GPs' health and patient care, doctors warn. British Medical Journal Publishing Group; 2021.



For peer review only

3. NHS E. The NHS Long Term Plan 2019 [Available from: https://www.longtermplan.nhs.uk/wp-content/uploads/2019/08/nhs-long-term-plan-version-1.2.pdf.

4. Park S, Leedham-Green K, Cohen T. What is generalism and why is it important? In: Park S, Leedham-Green K, editors. Generalism in Clinical Practice and Education: UCL Press 2024.

5. Hodes S, Hussain S, Jha N, Toberty L, Welch E. If general practice fails, The NHS fails: thebmjopinion 2021 [Available from: https://blogs.bmj.com/bmj/2021/05/14/if-general-practice-fails-the-nhs-fails/.

6. Hann M, Reeves D, Sibbald B. Relationships between job satisfaction, intentions to leave family practice and actually leaving among family physicians in England. The European Journal of Public Health. 2011;21(4):499-503.

7. Long L, Moore D, Robinson S, Sansom A, Aylward A, Fletcher E, et al. Understanding why primary care doctors leave direct patient care: a systematic review of qualitative research. BMJ Open. 2020;10(5):e029846.

8. Mol A. The logic of care: Health and the problem of patient choice: Routledge; 2008.

9. BMA. Pressures in general practice data analysis 2023 [Available from: https://www.bma.org.uk/advice-and-support/nhs-delivery-and-workforce/pressures/pressures-in-general-practice-data-analysis.

10. Cox J, Clark C, Sanders T. Rural healthcare: CRC Press; 2023.

11. Beech J, Fraser C, Gardner T, Buzelli L, Williamson S, Alderwick H. Stressed and overworked: what the Commonwealth Fund’s 2022 International Health Policy Survey of Primary Care Physicians in 10 Countries means for the UK. Health Foundation. Mar 2023.

12. NHS. Appointments in General Practice, October 2023 [Available from: https://digital.nhs.uk/data-and-information/publications/statistical/appointments-in-general-practice/october-2023#.

13. Hobbs FDR, Bankhead C, Mukhtar T, Stevens S, Perera-Salazar R, Holt T, et al. Clinical workload in UK primary care: a retrospective analysis of 100 million consultations in England, 2007-14. Lancet. 2016;387(10035):2323-30.

14. Sinnott C, Moxey JM, Marjanovic S, Leach B, Hocking L, Ball S, et al. Identifying how GPs spend their time and the obstacles they face: a mixed-methods study. British Journal of General Practice. 2022;72(715):e148-e60.

15. Khan N. ‘Hidden’no more: the real work of the ‘part-time’GPs working full-time hours. British Journal of General Practice. 2023;73(726):26-7.

16. Freeman G, Hughes, J. Continuity of care and the patient experience 2010 [Available from: https://www.kingsfund.org.uk/sites/default/files/field/field_document/continuity-care-patient-experience-gp-inquiry-research-paper-mar11.pdf.

17. Sandvik H, Hetlevik, O, Blinkenberg, J, Hunskaar, S. Continuity in general practice as predictor of mortality, acute hospitalisation, and use of out-of-hours care: a registry-based observational study in Norway. British Journal of General Practice. 2022;72(715).

18. Kajaria-Montag H, Freeman M, Scholtes S. Continuity of care increases physician productivity in primary care: INSEAD; 2022.

19. Laurant M, Harmsen M, Wollersheim H, Grol R, Faber M, Sibbald B. The impact of nonphysician clinicians. Medical Care Research and Review. 2009;66(6_suppl):36S-89S.

20. Cadogan SL, Browne JP, Bradley CP, Fitzgerald AP, Cahill MR. Physician and practice characteristics associated with immunoglobulin test ordering. Family Practice. 2018;35(1):41-6.

21. Morgan S, Henderson KM, Tapley A, Scott J, Van Driel ML, Spike NA, et al. Pathology test-ordering behaviour of Australian general practice trainees: a cross-sectional analysis. International Journal for Quality in Health Care. 2015;27(6):528-35.

For peer review only

22. Grigoroglou C, Walshe K, Kontopantelis E, Ferguson J, Stringer G, Ashcroft DM, et al. Locum doctor use in English general practice: analysis of routinely collected workforce data 2017–2020. British journal of general practice. 2022;72(715):e108-e17.
23. Nussbaum C, Massou E, Fisher R, Morciano M, Harmer R, Ford J. Inequalities in the distribution of the general practice workforce in England: a practice-level longitudinal analysis. BJGP Open. 2021;5(5).
24. Pawson R. Evidence-Based Policy: A Realist Perspective London: SAGE Publications.; 2006.
25. Marchand C, Peckham S. Addressing the crisis of GP recruitment and retention: a systematic review. Br J Gen Pract. 2017;67(657):e227-e37.
26. Andah E, Essang B, Friend C, Greenley S, Harvey K, Spears M, et al. Understanding the impact of professional motivation on the workforce crisis in medicine: a rapid review. BJGP Open. 2021;5(2).
27. Verma P, Ford JA, Stuart A, Howe A, Everington S, Steel N. A systematic review of strategies to recruit and retain primary care doctors. BMC Health Serv Res. 2016;16:126.
28. Lachish S, Svirko E, Goldacre MJ, Lambert T. Factors associated with less-than-full-time working in medical practice: results of surveys of five cohorts of UK doctors, 10 years after graduation. Human resources for health. 2016;14:1-11.
29. Fletcher E, Abel GA, Anderson R, Richards SH, Salisbury C, Dean SG, et al. Quitting patient care and career break intentions among general practitioners in South West England: findings of a census survey of general practitioners. BMJ open. 2017;7(4):e015853.
30. Dale J, Potter R, Owen K, Leach J. The general practitioner workforce crisis in England: a qualitative study of how appraisal and revalidation are contributing to intentions to leave practice. BMC family practice. 2016;17:1-7.
31. Doran N, Fox F, Rodham K, Taylor G, Harris M. Lost to the NHS: a mixed methods study of why GPs leave practice early in England. British journal of general practice. 2016;66(643):e128-e35.
32. Smith F, Goldacre MJ, Lambert TW. Retirement ages of senior UK doctors: national surveys of the medical graduates of 1974 and 1977. BMJ open. 2018;8(6):e022475.
33. Campbell JL, Fletcher E, Abel G, Anderson R, Chilvers R, Dean SG, et al. Policies and strategies to retain and support the return of experienced GPs in direct patient care: the ReGROUP mixed-methods study. 2019;7:1-288.
34. Park S, Berlin A. 'Divided we fail': understanding inter-relations and the role of the generalist. British Journal of General Practice. 2012;62(604):596-8.
35. Bansal A, Greenley S, Mitchell C, Park S, Shearn K, Reeve J. Optimising planned medical education strategies to develop learners' person-centredness: A realist review. Medical Education. 2022;56(5):489-503.
36. Graham A. Written evidence submitted by Dr Anna Graham PhD MRCP MRCGP (FGP0130) 2021 [Available from: https://committees.parliament.uk/writtenevidence/41518/pdf/.
37. Pereira Gray DJ, Sidaway-Lee K, White E, Thorne A, Evans PH. Continuity of care with doctors-a matter of life and death? A systematic review of continuity of care and mortality. BMJ Open. 2018;8(6):e021161.
38. Abrams R, Wong G, Mahtani KR, Tierney S, Boylan A-M, Roberts N, et al. Delegating home visits in general practice: a realist review on the impact on GP workload and patient care. British Journal of General Practice. 2020;70(695):e412-e20.
39. Bienkowska-Gibbs T, King S, Saunders C, Henham M. New organisational models of primary care to meet the future needs of the NHS: a brief overview of recent reports. RAND EUROPE 2015 2015.

For peer review only

527 40. Papoutsi C, Mattick K, Pearson M, Brennan N, Briscoe S, Wong G. Interventions to improve antimicrobial prescribing of doctors in training (IMPACT): a realist review. 2021.

529

For peer review only

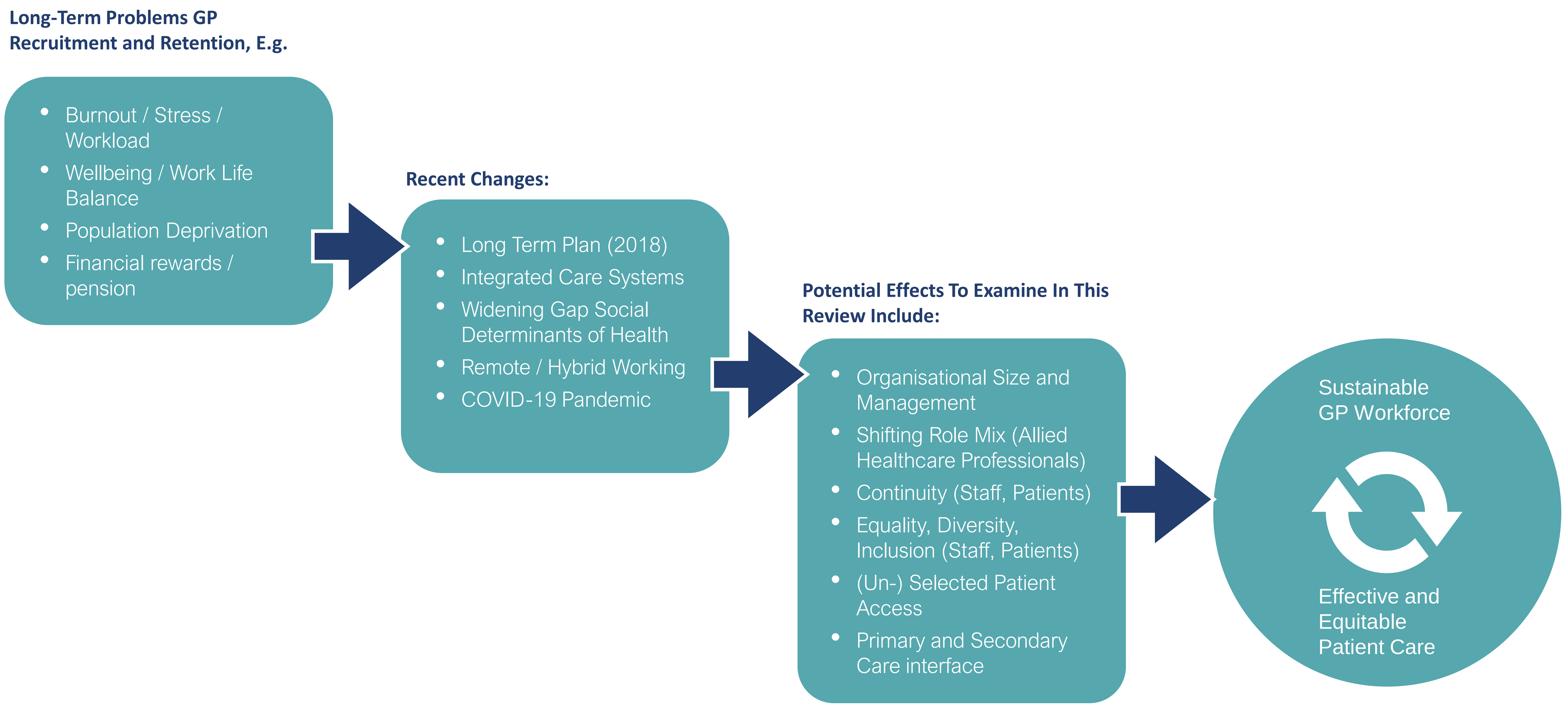
Long-Term Problems GP Recruitment and Retention, E.g.
Burnout / Stress / Workload
Wellbeing / Work Life Balance
Population Deprivation
Financial rewards / pension
Recent Changes:
Long Term Plan (2018)
Integrated Care Systems
Widening Gap Social Determinants of Health
Remote / Hybrid Working
COVID-19 Pandemic
Potential Effects To Examine In This Review Include:
Organisational Size and Management
Shifting Role Mix (Allied Healthcare Professionals)
Continuity (Staff, Patients)
Equality, Diversity, Inclusion (Staff, Patients)
(Un-) Selected Patient Access
Primary and Secondary Care interface
Sustainable GP Workforce
Effective and Equitable Patient Care

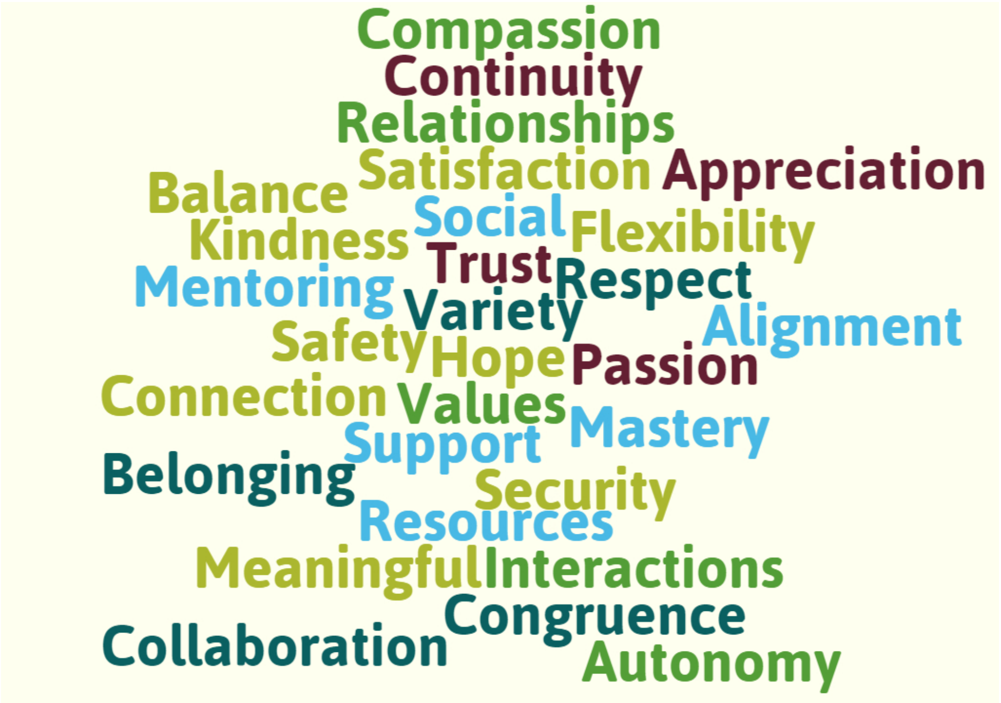


Figure 2: Word cloud visualisation.

361x253mm (72 x 72 DPI)

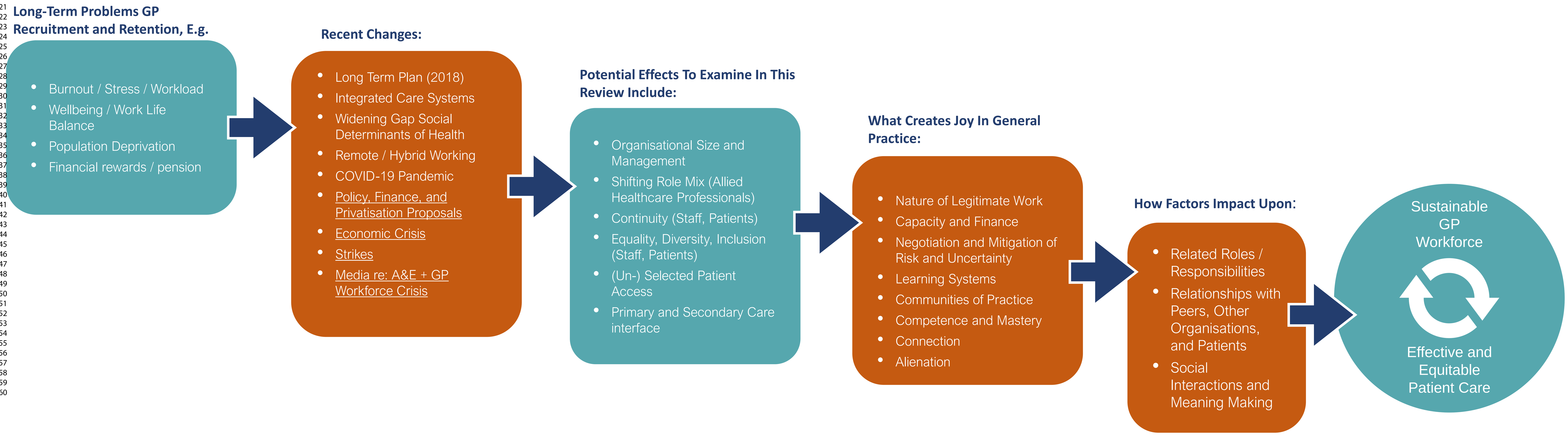
Long-Term Problems GP Recruitment and Retention, E.g.
Burnout / Stress / Workload
Wellbeing / Work Life Balance
Population Deprivation
Financial rewards / pension
Recent Changes:
Long Term Plan (2018)
Integrated Care Systems
Widening Gap Social Determinants of Health
Remote / Hybrid Working
COVID-19 Pandemic
Policy, Finance, and Privatisation Proposals
Economic Crisis
Strikes
Media re: A&E + GP Workforce Crisis
Potential Effects To Examine In This Review Include:
Organisational Size and Management
Shifting Role Mix (Allied Healthcare Professionals)
Continuity (Staff, Patients)
Equality, Diversity, Inclusion (Staff, Patients)
(Un-) Selected Patient Access
Primary and Secondary Care interface
What Creates Joy In General Practice:
Nature of Legitimate Work
Capacity and Finance
Negotiation and Mitigation of Risk and Uncertainty
Learning Systems
Communities of Practice
Competence and Mastery
Connection
Alienation
How Factors Impact Upon:
Related Roles / Responsibilities
Relationships with Peers, Other Organisations, and Patients
Social Interactions and Meaning Making
Sustainable GP Workforce
Effective and Equitable Patient Care

# Supplementary File 1

## Details of the search developed for MEDLINE:

| | | | |
|---|---|---|---|
| **Data Source** | Database | MEDLINE | |
| | Details | MEDLINE® Epub Ahead of Print, In-Process & Other Non-Indexed Citations, Ovid MEDLINE® Daily and Ovid MEDLINE® 1946 to present | |
| | Host | Ovid | |
| | Date searched | 20/04/2023 | |
| | Database Update | Daily update | |
| **Limiters** | English only? | Yes | |
| | Time period searched | 2013 - Present | |
| | Publications types | No limit | |
| | Other | | |
| **Results** | Items found | 504 | |
| | Internal duplicates (within one database) | 6 | |
| | External duplicates (between databases) | 0 | |
| | New | 498 | |
| In Ovid, save your search. Go to **Saved Searches** and click on the eye icon of your SR saved search to **View** your search. Copy and paste your search into **Notepad;** put tabs between the line number and text. Copy and paste into cell **B15**. | **Paste text of search strategy below** | | |
| | **Saved: Sustainable GP V6 FINAL MEDLINE** | | |
| | Medline (Ovid MEDLINE® Epub Ahead of Print, In-Process & Other Non-Indexed Citations, Ovid MEDLINE® Daily and Ovid MEDLINE®) 1946 to present | | |
| | | | |
| | 1 | general practi*.ti,ab,kw. | 92437 |
| | 2 | *General Practitioners/ | 8488 |
| | 3 | exp *General Practice/ | 51683 |
| | 4 | or/1-3 | 121611 |
| | 5 | exp *Work/ | 43272 |
| | 6 | exp *Workplace/ | 14,019 |
| | 7 | *Job Satisfaction/ | 12818 |
| | 8 | exp *Occupational Stress/ | 16249 |
| | 9 | exp *"Personnel Staffing and Scheduling"/ | 24174 |
| | 10 | (work* or staffing or career* or job*).ti,ab,kw. | 2041372 |
| | 11 | (stress* or burn out or burnout).ti,ab,kw. | 1066414 |
| | 12 | or/5-11 | 3013398 |
| | 13 | exp *Workforce/ | 7820 |
| | 14 | *Retirement/ | 6784 |
| | 15 | *Personnel Turnover/ | 3003 |
| | 16 | *Work Engagement/ | 559 |
| | 17 | *Career Mobility/ | 6434 |
| | 18 | (workforce* or staff* level*).ti,ab,kw. | 37788 |
| | 19 | (part?time* or "less than full time" or LTFT).ti,ab,kw. | 239 |
| | 20 | (retire* or retention* or retain* or sustain* or stability or turnover).ti,ab,kw. | 1509240 |
| | 21 | (work* adj1 pattern*).ti,ab,kw. | 1433 |

| | | |
|---|---|---|
| 22 | (flexible* adj1 work*).ti,ab,kw. | 1028 |
| 23 | ((reason* or rationale* or decision* or intention*) adj1 (leav* or stay*)).ti,ab,kw. | 313 |
| 24 | (career* adj1 (break* or end*)).ti,ab,kw. | 312 |
| 25 | ((participat* or engag*) adj1 (work* or job*)).ti,ab,kw. | 6597 |
| 26 | or/13-25 | 1560462 |
| 27 | 4 and 12 and 26 | 2544 |
| 28 | exp United Kingdom/ | 389181 |
| 29 | (national health service* or nhs*).ti,ab,in. | 265557 |
| 30 | (english not ((published or publication* or translat* or written or language* or speak* or literature* or citation*) adj5 english)).ti,ab. | 48488 |
| 31 | (gb or "g.b." or britain* or (british* not "british columbia") or uk or "u.k." or united kingdom* or (england* not "new england") or northern ireland* or northern irish* or scotland* or scottish* or ((wales or "south wales") not "new south wales") or welsh*).ti,ab,jw,in. | 2421175 |
| 32 | (bath or "bath's" or ((birmingham not alabama*) or ("birmingham's" not alabama*) or bradford or "bradford's" or brighton or "brighton's" or bristol or "bristol's" or carlisle* or "carlisle's" or (cambridge not (massachusetts* or boston* or harvard*)) or ("cambridge's" not (massachusetts* or boston* or harvard*)) or (canterbury not zealand*) or ("canterbury's" not zealand*) or chelmsford or "chelmsford's" or chester or "chester's" or chichester or "chichester's" or coventry or "coventry's" or derby or "derby's" or (durham not (carolina* or nc)) or ("durham's" not (carolina* or nc)) or ely or "ely's" or exeter or "exeter's" or gloucester or "gloucester's" or hereford or "hereford's" or hull or "hull's" or lancaster or "lancaster's" or leeds* or leicester or "leicester's" or (lincoln not nebraska*) or ("lincoln's" not nebraska*) or (liverpool not (new south wales* or nsw)) or ("liverpool's" not (new south wales* or nsw)) or ((london not (ontario* or ont or toronto*)) or ("london's" not (ontario* or ont or toronto)) or manchester or "manchester's" or (newcastle not (new south wales* or nsw)) or ("newcastle's" not (new south wales* or nsw)) or norwich or "norwich's" or nottingham or "nottingham's" or oxford or "oxford's" or peterborough or "peterborough's" or plymouth or "plymouth's" or portsmouth or "portsmouth's" or preston or "preston's" or ripon or "ripon's" or salford or "salford's" or salisbury or "salisbury's" or sheffield or "sheffield's" or southampton or "southampton's" or st ablans or stoke or "stoke's" or sunderland or "sunderland's" or truro or "truro's" or wakefield or "wakefield's" or wells or westminster or "westminster's" or winchester or "winchester's" or wolverhampton or "wolverhampton's" or (worcester not (massachusetts* or boston* or harvard*)) or ("worcester's" not (massachusetts* or boston* or harvard*)) or (york not ("new york" or ny or ontario* or ont or toronto*)) or ("york's" not ("new york" or ny or ontario* or ont or toronto*))))).ti,ab,in. | 1720005 |
| 33 | (bangor* or "bangor's" or cardiff or "cardiff's" or newport or "newport's" or st asaph or "st asaph's" or st davids or swansea or "swansea's").ti,ab,in. | 69203 |
| 34 | (aberdeen or "aberdeen's" or dundee or "dundee's" or edinburgh or "edinburgh's" or glasgow or "glasgow's" or inverness or (perth not australia*) or | 253603 |
| 35 | "newry's").ti,ab,in. | 33231 |
| 36 | or/28-35 | 3037247 |
| 37 | (exp africa/ or exp americas/ or exp antarctic regions/ or exp arctic regions/ or exp asia/ or exp oceania/) not (exp United Kingdom/ or europe/) | 3308914 |
| 38 | 36 not 37 | 2875391 |
| 39 | 27 and 38 | 793 |
| 40 | limit 39 to (english language and yr="2013 -Current") | 504 |

BMJ Open

# General practitioner workforce sustainability to maximise effective and equitable patient care: a realist review protocol.

| | |
|---|---|
| Journal: | *BMJ Open* |
| Manuscript ID | bmjopen-2023-075189.R2 |
| Article Type: | Protocol |
| Date Submitted by the Author: | 04-Mar-2024 |
| Complete List of Authors: | Park, Sophie; University College London, Department of Primary Care and Population Health<br>Owen, Emily; University College London, Department of Primary Care & Population Health<br>Burford, Bryan; Newcastle University, School of Medicine<br>Cohen, Tanya; University College London, Department of Primary Care and Population Health<br>Duddy, Claire; University of Oxford, Nuffield Department of Primary Care Health Sciences<br>Dunn, Harry; University of Cambridge, School of Clinical Medicine<br>Fadia , Vacha ; University College London, Department of Primary Care & Population Health<br>Goodman, Claire; University of Hertfordshire, Centre for Research in Public Health and Community Care<br>Henry , Cecily ; University College London, Department of Primary Care and Population Health<br>Lamb , Elizabeth; Newcastle University, School of Medicine<br>Ogden , Margaret; University College London, Department of Primary Care and Population Health<br>Rapley, Tim; Northumbria University, Department of Social Work, Education and Community Wellbeing<br>Rees, Eliot; University College London, Department of Primary Care and Population Health; Keele University, School of Medicine<br>Vance, Gillian ; Newcastle University, School of Medicine<br>Wong, Geoff; University of Oxford, Nuffield Department of Primary Care Health Sciences |
| <b>Primary Subject Heading</b>: | General practice / Family practice |
| Secondary Subject Heading: | Health services research, Sociology, Public health, Health policy |
| Keywords: | Health Equity, Organisation of health services < HEALTH SERVICES ADMINISTRATION & MANAGEMENT, Patient-Centered Care, Physicians, Primary Care < Primary Health Care |
| | |

SCHOLARONE™
Manuscripts

RUNNING HEAD: REALIST REVIEW PROTOCOL

# General practitioner workforce sustainability to maximise effective and equitable patient care: a realist review protocol.

Sophie Park[1], Emily C. Owen[1], Bryan Burford[2], Tanya Cohen[1], Claire Duddy[3], Harry Dunn[4], Vacha Fadia[1], Claire Goodman[5], Cecily Henry[1], Elizabeth I. Lamb[2], Margaret Ogden[1], Tim Rapley[6], Eliot L. Rees[1,7], Gillian Vance[2], Geoff Wong[3]

[1]Department of Primary Care and Population Health, University College London, London, UK, [2] School of Medicine, Newcastle University, Newcastle Upon Tyne, UK, [3]Nuffield Department of Primary Care Health Sciences, University of Oxford, Oxford, UK, [4]School of Clinical Medicine, University of Cambridge, Cambridge, UK, [5]Centre for Research in Public Health and Community Care, University of Hertfordshire, Hatfield, UK, [6]Department of Social Work, Education and Community Wellbeing, Northumbria University, Newcastle Upon Tyne, UK, [7]School of Medicine, Keele University, Keele, UK.

Corresponding author:

Dr Emily C. Owen; UCL Research Department of Primary Care & Population Health; Royal Free Campus; Hampstead, London; NW3 2PY.

Email: Emily.owen@ucl.ac.uk; Phone: 020 7794 0500.

Keywords: General Practice; Workforce; Sustainability; Healthcare system; Retention.

Word count: 6028.

For peer review only

ABSTRACT

**Introduction:** There are not enough General Practitioners (GPs) in the UK National Health Service. This problem is worse in areas of the country where poverty and underinvestment in health and social care mean patients experience poorer health compared with wealthier regions. Encouraging more doctors to choose and continue in a GP career is a government priority. This review will examine which aspects of the healthcare system affect GP workforce sustainability, how, why, and for whom.

**Methods and analysis:** A realist review is a theory-driven interpretive approach to evidence synthesis, that brings together qualitative, quantitative, mixed-methods research, and grey literature. We will use a realist approach to synthesise data from the available published literature to refine an evidence-based programme theory that will identify the important contextual factors and underlying mechanisms that underpin observed outcomes relating to GP workforce sustainability. Our review will follow Pawson's five iterative stages: (1) finding existing theories; (2) searching for evidence; (3) article selection; (4) data extraction; and (5) synthesising evidence and drawing conclusions. We will work closely with key stakeholders and embed patient and public involvement throughout the review process to refine the focus of the review and enhance the impact and relevance of our research.

**Ethics and dissemination:** This review does not require formal ethical approval as it draws on secondary data from published articles and grey literature. Findings will be disseminated through multiple channels, including publication in peer-reviewed journals, at national and international conferences, and other digital scholarly communication tools such as video summaries, X, and blog posts.

**PROSPERO registration number**: CRD42023395583.

For peer review only

Strengths and limitations of this study:

- This is the first systematic review to use realist methodology to explore which aspects of the healthcare system affect GP workforce sustainability, how, why, and for whom.
- We will include a diverse range of evidence, including, empirical articles, conference materials, policy reports, and editorials, increasing review depth and breadth.
- We will embed patient and public involvement and stakeholders throughout the design, analysis, and report stages of the project, enhancing impact and relevance.
- We will only include documents that are written or translated in English.
- Our context-mechanism-outcome configurations and refined programme theory may be limited by the availability, quality, and richness of existing identified literature in this field.

For peer review only

# INTRODUCTION

UK general practice has been described as being in crisis (1). A shortage and exodus (2) of General Practitioners (GPs) is an urgent and challenging problem, attracting significant media attention and widespread public debate. The National Health Service (NHS) Long Term Plan proposed a net increase of 5000 GPs, alongside expectations of work distribution and re-allocation across primary care teams (3). The impact of these changes on the GP workforce remains relatively unexplored. In the UK, general practice provides comprehensive (a patient may present with any type of problem), person-centred (holistic caring and individualised care), and universal (everyone can attend) access to healthcare. The ways in which this care is organised, and delivered do tend, therefore, to differ depending on the needs of local communities, available resources, and capacity (4).

It is widely acknowledged that general practice provides 90% of patient care utilising approximately 8% of the NHS budget (5), meaning this is a key element of broader debates about a whole-system NHS moral and financial crisis. Existing research has focused on 'numbers in' 'numbers out' calculations of workforce need, predictions, and requirements. GPs' decisions to stay or leave are often positioned as an individual 'choice' (6, 7, 8), resilience, or failure, with less attention given to the social and organisational milieu in which those decisions are produced. A systems approach recognises the need to acknowledge this inherent dynamic complexity, by studying the interconnected components (e.g. the nature of social interactions between patients, colleagues, and external institutions, alignment of personal and policy priorities, and organisational support structures) that work together in an integrated and coordinated way to sustain and enhance the GP workforce.

The figures reflecting shifts in the number of GPs available are well-rehearsed. For example, as of February 2023, the number of full-time equivalent GPs has fallen significantly

For peer review only

(approximately 2,100 fewer fully qualified full-time GPs compared to September 2015) (9), many GPs are approaching traditional retirement ages, and the challenges of recruiting and retaining GPs in rural and coastal areas is well recognised, both nationally and internationally (10). UK GPs have reported higher levels of emotional distress than GPs in nearly all other countries (11). In parallel, readily measurable elements of GP work have been reported to increase, with 37.2 million consultations estimated to have been delivered by practices and primary care networks in October 2023, of which 2.9 million were COVID-19 vaccinations (12). Consultations are reported to have increased in length (13), alongside changes in form (e.g. use of technology). Measures of GP time spent on operational challenges and problems are high (14) and the 'hidden', non-patient-facing work of GPs is increasingly being recognised (15). It is widely acknowledged that GPs staying in practice has benefits for both patient care (16, 17), and practice productivity (18). For example, experienced GPs work more efficiently, lessening the scale of patient medicalisation, investigation, and treatment, and requiring shorter appointment times compared to trainees and other healthcare professionals (19, 20, 21). We can also see that practices with a higher proportion of patients in deprived areas; lower care quality commission ratings; single-handed and rural practices, have fewer GPs overall and more locums (22) emphasising the importance of wider contextual factors in shaping the nature of work and workforce patterns (23).

A systems approach is well suited as a basis for examining the multitude of factors influencing the GP workforce and how these ultimately shape career decisions. By taking a system and theory-driven realist approach to understanding what works, in which contextual circumstances, and to what extent, we aim to make visible the often unsaid or implicit issues that influence working environments, relationships, and organisational culture. The findings from our review will offer structural and organisational recommendations to inform sustainable, context-specific ways of future working, which can underpin effective and

For peer review only

equitable patient care. For the purposes of our review, we define equitable patient care as the process through which healthcare systems maximise inclusive ways to facilitate access and care delivery for patients. While equality implies delivery of the same for all, equity focuses on ensuring patients have the best opportunity to reach needed outcomes, even if systems must change or adapt processes, to enable this. Effective patient care may vary according to the patient's needs and contextual circumstances but will include the best possible fit for them at that particular point in time (4).

## METHODS

### Review aim, research objectives, and research questions

### Aim

This review aims to examine which aspects of the healthcare system affect GP workforce sustainability, how, why, and for whom.

### Research Objectives:

1. Develop a programme theory through an evidence synthesis of how the GP workforce can be sustainable and work effectively with others to support effective and equitable patient care.

2. Embed and respond to patient and public involvement (PPI) and stakeholder perspectives throughout the design, analysis, and report stages of the project, thus maximising the relevance and utility of review findings.

For peer review only

3. Make recommendations for practice and policy based on the refined programme theory.

**Research Questions:**

What can we learn from the existing literature that will promote GP workforce sustainability, to support effective and equitable patient care? Specifically:

1. Which mechanisms produce intended and/or unintended outcomes in GP workforce sustainability?

2. What are the important contexts which influence whether different mechanisms produce intended and/or unintended outcomes in GP workforce sustainability?

3. Which interventional strategies lead to intended and/or unintended outcomes for GP workforce sustainability?

Approach

We will conduct a realist review, synthesising data from the published literature to produce a refined evidence-based programme theory. A programme theory explains how a programme, intervention, or process works (e.g. how we can develop a more sustainable GP workforce) (24). The programme theory will identify the important contexts (conditions or circumstances) and mechanisms (underlying causal forces) which underpin observed outcomes (intended and unintended) relating to GP workforce sustainability to support the delivery of effective and equitable patient care. A realist review is an interpretive and theory-driven approach to evidence synthesis, that brings together data drawn from quantitative, qualitative, and mixed methods research, as well as the wider grey literature (25). Using this data and a realist logic of analysis will allow us to examine diverse evidence with a clear

For peer review only

focus on understanding factors which support or challenge GP workforce sustainability, how these are shaped by important contexts, and the mechanisms that underpin them. Based on this understanding, captured in our final programme theory, we will be able to identify and prioritise important system-level contexts that may be amenable to change. Our review began in January 2023 and will end December 2025.

Initial scoping searches using EMBASE, MEDLINE and PsycINFO identified a range of qualitative and quantitative primary and secondary evidence focused on GP retention. We have identified four existing reviews focused on GP retention (7, 26, 27, 28), and four additional studies from broader scoping searches, including one examining factors associated with part-time working (29). Existing studies on this topic are often descriptive, identifying a complex array of factors associated with workforce attrition without theoretical analysis. These include individual issues (e.g. wellbeing, burnout, identity), practice (e.g. workload, administrative burden, clinical autonomy (26) - and opportunities for relational continuity of care (7, 27), and national (e.g. revalidation) contexts (1, 30, 31, 32). One study found the intrinsic attractions of retirement (e.g. increased time for leisure') contributed to GPs leaving practice (33). Factors shaping GP retention in one UK region (34) were described as 'push' (workload, stress, job dissatisfaction, and organisational injustice de-motivating factors) and 'pull' (work-life balance).

This review will attend to the complex systems in which GPs work, in order to better understand the factors which, keep, sustain, and enable GPs to flourish within their work. A realist approach will allow us to examine a wide range of literature about the system and related social interactions (e.g. across the primary and secondary care interface, between patients and clinicians), and to integrate patient and stakeholder experience to inform our approach and produce new ways of thinking about this challenging field. We will identify

For peer review only

causal explanations to illuminate the connections between GP work (e.g. nature and scope) and GP career intentions. Depending on the available evidence, we aim to examine a range of contexts (e.g. location, employment arrangements such as small or large institutions, salaried, partnered, payment by performance), systems of support inside or outside practices, and patient deprivation. We will examine the relationships between these contextual factors and a range of reported outcomes. This will help to identify the circumstances in which a sustainable and flourishing GP workforce for delivery of effective and equitable patient care can be realised.

Our approach will follow Pawson's (25) five iterative stages for realist reviews detailed below. This begins with an initial programme theory developed through scoping searches and preliminary consultation with stakeholders and patients. As the review progresses, we will gather and interpret data from the literature to refine and develop our explanations of important outcomes, drawing on the expertise of our PPI and stakeholder groups. At the end of the project, our final programme theory will consist of evidenced context-mechanism-outcome configurations (CMOCs), presenting realist causal explanations for outcomes relating to the GP workforce sustainability to support effective and equitable patient care.

**Patient and Public Involvement**

We have three PPI co-applicants within our core research team. The team also includes professionals from a range of disciplinary backgrounds including general practice, secondary care, nursing, psychology, and sociology. Additionally, we have recruited six PPI contributors from coastal, rural, and diverse urban contexts. An equality of opportunity approach will be taken throughout this process, enabling us to respond in an agile way to maximise PPI involvement. For example, helping to refine the programme theory, shape our findings and contribute to both the format and content of our outputs (e.g. written word,

For peer review only

infographics), and ensuring that proposed solutions are both feasible and acceptable. Finally, our PPI members will play an essential role in developing our dissemination strategy, and the format and content of the outputs of this project. We also have a stakeholder group including professionals representing a variety of GP employment models, paramedic, pharmacy, policy, practice, management, and educational backgrounds.

In the development of this proposal, we invited PPI and stakeholders to share their priorities. GPs often work at the interface between human experience and the technicalities of pathology, negotiating boundaries and overlaps between stress, illness, and disease (35). Person-centred care has been recognised as a key element of this process (36, 37). Knowledge of the 'person' can be established over time, building on trust and relational currency between patient and practitioner (38). One key area of concern in PPI and stakeholder discussions during development of this review protocol, was 'care of other'. GPs spoke about the importance of person-centred care, while PPI co-applicants highlighted the importance of looking after practitioners and supporting their well-being. As one PPI contributor explained, if patients are treated as 'a number' within the healthcare system, this is potentially mirrored in their treatment and expectation of healthcare professionals. One key area about GP workforce sustainability in this review, therefore, is the relational elements of GP work, and how these may support or undermine workforce sustainability.

We invited PPI and stakeholders to help produce three case study examples about GP workforce sustainability:

**Case Study 1.** In the UK, following the introduction of the NHS Long Term Plan, multi-disciplinary team working in general practice is increasing (3). Many practices now employ pharmacists, paramedics, physician associates, physiotherapists, and mental health professionals. In some circumstances, this has enabled GPs to focus on the clinical care of

For peer review only

particularly 'complex' patients (e.g. with multimorbidity), delegate some home visits (39), and prescribing demands (40). Yet, some stakeholders report a) anecdotal increases in workload relating to supervision and support of new staff and b) increased levels of follow up (e.g. lower thresholds for initiating patient investigations), c) patients needing to attend multiple appointments to resolve a problem(s) and d) challenges negotiating access for patients with unselected and comprehensive healthcare problems to see the most appropriate professional, at the most appropriate time. These fast-moving changes to the GP role and patient access to GP care, have therefore been experienced both as producing opportunities and challenges.

**Case Study 2.** Relationships between GPs and secondary care colleagues can influence patient care and access to services. For example, if close and rapid communication is established (e.g. informal sharing of numbers, or formal routes for email advice and guidance), GPs can progress patient care, minimising referrals and waiting list delays, and maximising timely patient care delivery in the primary care setting. This could have an impact on a GP's sense of agency or autonomy to provide immediate care for patients, but also a sense of working as part of a distributed team, rather than alone. As an example: a specialist nurse managing a Paediatric Allergy clinic advice service. In one area, this enables access to advice regarding initial investigations and treatment. In another area, this increases recommendations to refer. This means waiting lists could be shortened or lengthened, expediting, or delaying assessment of urgent or severe patients, or periods of time requiring additional GP support.

**Case Study 3.** GPs sometimes develop 'special interests' (e.g. supporting patients with mental health problems). This can be positive for practices, patients, and GP professional development. Yet, it can also produce challenges and inequalities if not managed well. For

For peer review only

example, patients requiring more clinical input, longer consultations and emotional investment being directed to seek help from particular GPs. Without adequate planning, this can result in GPs who support these patients running late, working longer hours (for no extra pay) to complete other routine tasks. Without sufficient resources and support, this can affect GP wellbeing and potential burnout.

Step 1: Locating Existing Theories

We have developed an initial programme theory to describe our assumptions about important influences on GP workforce sustainability. This outlines potentially important contexts, active mechanisms, and outcomes relating to GP workforce sustainability that we need to consider or build upon over the course of the review. Important contexts, for example, might include organisation size, employment type, practice skill-mix, and nature of patient contact. Mechanisms could comprise continuity, including, for instance, relational, informational, longitudinal, and management types of continuity (41, 42), peer dialogue, or clinical autonomy. A draft of our initial programme theory is provided in Figure 1.

**Figure 1** Initial programme theory – used at the beginning of realist review projects to map initial explanatory theories.

****Insert Figure 1 Around Here****

**Engaging PPI and stakeholder perspectives in expanding the initial programme theory: Exploring how to create joy.**

To expand our initial programme theory and shape initial searches, we carefully planned a series of individual and small group discussions with PPI and stakeholder members to open a

For peer review only

conversation about ‘what creates joy in general practice and enables (or produces conditions for) ‘equitable and effective patient care’. We focused on joy (and meaning making) as most of the literature we identified in our initial scoping searches was predominantly negative, highlighting the reasons why GPs leave or consider leaving direct patient care. We recognise that many GPs continue to enjoy and derive pleasure from their work (43). As such, our aim throughout the PPI and stakeholder discussions was to understand the factors that keep GPs engaged meaningfully in their work. Participants were invited to draw on their own experiences and perspectives to explore this topic. Following introductions, we asked our PPI and stakeholder members to draw, describe, or write words which represented their thoughts, feelings or experiences about creating joy in general practice and enabling effective and equitable patient care. These were probed and discussed to build up a map of issues, including system level factors (e.g. patient access, nature of consultation interactions, interface between team members and other organisations, explicit patient facing work, and other ‘hidden’ work such as administration tasks). This map helped to modify and expand our initial programme theory, which was then used to shape initial searches and became the starting point for our review. An overview of the key concepts from our PPI and stakeholder discussions can be found in our word cloud (Figure 2). A draft of our expanded initial programme theory is provided in Figure 3.

**Figure 2:** Word cloud visualisation

****Insert Figure 2 Around Here****

**Figure 3:** Expanded initial programme theory after stakeholder and PPI consultation workshops.

For peer review only

****Insert Figure 3 Around Here****

Step 2: Searching for Evidence

We will conduct searches to assemble a relevant body of literature that contains data we can use to develop and refine the initial programme theory developed in Step 1. To help us work efficiently, we will re-use search strategies employed in our scoping review and extend and update these to identify new, relevant material also indexed in additional databases (CINAHL, HMIC, Web of Science - SCI-EXPANDED and SSCI indexes). The main databases we will search include: MEDLINE; Embase; PsychINFO; CINAHL; HMIC; and Web of Science. These searches will combine free text and subject heading (MeSH) terms for GPs with a range of terms relating to workforce and retention outcomes. The full details of the search developed for MEDLINE are available in online supplementary file 1. We will conduct searches from other data sources including relevant organisational websites (e.g. Health Foundation, King's Fund, RCGP, DHSC) to identify grey literature.

We will limit our searches to identify material published in the English language. No limits will be placed on study design, as all relevant evidence (including opinion and commentary) can be used to inform programme theory development. As the review progresses, we will identify additional material via citation searching, our PPI and stakeholder members, and alerting services. Our initial inclusion criteria will include GP as the object of discussion or participant, discussion, or study of UK general practice setting (including out-of-hours), include enquiry or discussion of GP workforce and/or GP work (e.g. nature, role, change, shift), relationship between GP and work or meaning attributed to work, and documents published within the last ten years.

For peer review only

318 Throughout the review, discussions within the project team and with PPI and
319 stakeholders will be on-going, framing the focus and direction of literature selection,
320 extraction, and analysis, and informing the production of the final programme theory
321 configurations. At each stage, the team will ask 'is this relevant to the programme theory?',
322 focusing on the connection between 'GP workforce sustainability' and 'effective and
323 equitable patient care' to inform decisions about whether to explore and include certain areas
324 of evidence and literature.

325 If necessary, we will conduct additional searches later in the project, with the aim of
326 identifying data to fill gaps or refine specific aspects of our developing programme theory.
327 Our additional searches will be guided by the input of our PPI and stakeholder groups, as we
328 work with them to prioritise areas of the developing programme theory for exploration.
329 Crucially, we will explore key workforce sustainability concepts (see above) with our PPI
330 and stakeholders, alongside the United Nations definition of sustainable practice which
331 includes working environment, resources, and people interacting within that system (14).

332 Step 3: Article Selection

333 We will screen documents for inclusion in the review in a three-step process: screening by
334 title/abstract, following by screening in full text, with final selection resting on an assessment
335 of each document's relevance (whether it contains data relating to relevant contexts,
336 mechanisms, outcomes or the relationships between these) and rigour (whether the methods
337 used to generate each piece of data are credible and trustworthy) (25). To ensure consistency
338 in this process, a 10% sample of decisions made at each stage will be independently checked
339 by research team members (including CD, ER, BB and LL). Any discrepancies or
340 disagreements will be discussed with ECO and the research team, and an agreed resolution
341 documented.

For peer review only

Step 4: Data Extraction

Included full text documents will be uploaded to NVivo (qualitative data analysis software) for coding. Coding will be inductive (codes created to categorise data as reported in the included documents), deductive (some codes will be created in advance, based on the initial programme theory in Step 1) and retroductive (codes created based on an interpretation of the data, to infer what hidden causal forces may generate outcomes). Each new piece of relevant data identified will be used to refine and develop our programme theory and as the review progresses, included documents will be re-scrutinised to identify relevant data that may have been missed earlier, or as the programme theory developed. As with our screening and selection processes, a 10% sample of included documents will be independently checked by CD, ER, BB, and EL to ensure consistency. Any discrepancies or disagreements will be discussed within the research team and documented.

Step 5: Synthesising the Evidence and Drawing Conclusions

We will use a realist logic of analysis to make sense of the extracted data and develop CMOCs that offer explanations for outcomes relating to GP workforce sustainability and provision of effective, equitable patient care. We will use interpretive cross-case comparison to understand and explain how and why observed outcomes occur. For example, we will compare documents that describe contexts in which are reported to enable GPs to flourish and sustain their working roles, to understand how contexts influence GP workforce sustainability and support effective and equitable patient care.

For peer review only

We will use a proven analysis and synthesis process (44). In brief, to operationalise our realist analysis, we will ask the following questions about the included documents and data therein:

- Interpretation of meaning: do the documents provide data that may be interpreted as functioning as context(s), mechanism(s), or outcome(s)?
- Interpretations and judgements about CMOCs: what is the CMOC for the data that has been interpreted as functioning as context, mechanism, or outcome?
- Interpretations and judgements about programme theory: how does this CMOC relate to the initial programme theory?

At the end of the process, we will produce a refined programme theory, underpinned by the data extracted from included documents, on which our co-production activities will build for dissemination of review findings.

Ethics and Dissemination

Dissemination

Our PPI co-applicants and stakeholders will help us to decide on the content, storyboarding and format (e.g. websites, leaflets, videos, social media). In addition to the final report, we will produce:

1. **Academic outputs**: e.g. protocol publication. Findings (to be submitted to a high-impact, open-access, and peer-reviewed journal), as well as tailored papers to different disciplinary journals.

For peer review only

2. **Audience-specific practitioner** 'how to' publications which outline practical advice on ways to maximise the sustainability of the GP workforce to support effective and equitable patient care.

3. **User-friendly summaries** of the review findings tailored to the needs of different audiences including the public and service users.

Our dissemination strategy will build upon a participatory approach, embedding PPI co-applicants and stakeholder involvement throughout the development of this research and project timeline, including opportunities for co-authorship and co-presenting. On-going engagement with key stakeholders will maximise opportunities to utilise our established networks, communication channels and links to policymakers and providers. Our approach will be integrative, valuing the different forms of knowledge needed to produce findings capable of informing complex decision-making within policy and practice.

Our audiences are:

1. Policymakers, health system decision-makers, and commissioners who will be key to implementing recommendations from our review.
2. Providers and practitioners whom we will brief on our findings.
3. Members of the public and charities - for whom we will tailor press releases, social media posts and engage directly via our PPI and stakeholder networks. Our migrant PPI members will help devise ways to best disseminate our outputs to non-English speaking communities.

Ethics

Ethical approval is not required for this review as only secondary data sources will be used.

For peer review only

## DISCUSSION

### Importance of the Research

Following an escalation in changes affecting general practice during and after the COVID-19 pandemic, there are national and international debates about what general practice of the future should look like (45, 46, 47). Improving recruitment and retention of GPs should be a high priority in medical workforce planning. Yet, with many GPs reducing their working hours or retiring early, new theoretical perspectives and research is needed to examine how we can support and retain the GPs we have, alongside enabling recruitment of new GPs. We hope the findings from our review can influence political discussions and shape future patient care, contributing to the scholarship of the field, and to a more sustainable general practice. Throughout our research, we will generate new knowledge about the interdependencies between contextual factors, causal mechanisms, and outcomes of interest. The findings may be used to inform strategies and interventions intended to support, facilitate, and assist the GP workforce in delivering equitable and effective patient care. We will identify critical gaps in knowledge and prioritise the expectations for scope and nature of GP work.

### Authors' contributions

The realist review was conceptualised by SP. SP wrote the first draft of this protocol and it was reviewed and revised by ECO, BB, TC, CD, HD, VF, CG, CH, EIL, MO, TR, ELR, GV, and GW. The search strategy was developed by CD in consultation with ECO and the wider project team. All authors read and approved the final manuscript. SP is the guarantor.

### Funding Statement

For peer review only

This project is funded by the National Institute for Health Research (NIHR) School for Primary Care Research (SPCR593). Tim Rapley is a deputy theme lead within the NIHR Applied Research Collaboration Northeast and North Cumbria (NIHR200173). Emily Owen, Sophie Park, and Geoff Wong are members of the NIHR SPCR Evidence Synthesis Working Group. The views expressed are those of the authors and not necessarily those of the NIHR or the Department of Health and Social Care.

Competing interests

The authors have no conflicts of interests to declare.

## References

1. Owen K, Hopkins T, Shortland T, Dale J. GP retention in the UK: a worsening crisis. Findings from a cross-sectional survey. BMJ Open. 2019;9(2):e026048.
2. Iacobucci G. Burnout is harming GPs' health and patient care, doctors warn. British Medical Journal Publishing Group; 2021.
3. NHS E. The NHS Long Term Plan 2019 [Available from: https://www.longtermplan.nhs.uk/wp-content/uploads/2019/08/nhs-long-term-plan-version-1.2.pdf.
4. Park S, Leedham-Green K, Cohen T. What is generalism and why is it important? In: Park S, Leedham-Green K, editors. Generalism in Clinical Practice and Education: UCL Press 2024.
5. Hodes S, Hussain S, Jha N, Toberty L, Welch E. If general practice fails, The NHS fails: thebmjopinion 2021 [Available from: https://blogs.bmj.com/bmj/2021/05/14/if-general-practice-fails-the-nhs-fails/.
6. Hann M, Reeves D, Sibbald B. Relationships between job satisfaction, intentions to leave family practice and actually leaving among family physicians in England. The European Journal of Public Health. 2011;21(4):499-503.
7. Long L, Moore D, Robinson S, Sansom A, Aylward A, Fletcher E, et al. Understanding why primary care doctors leave direct patient care: a systematic review of qualitative research. BMJ Open. 2020;10(5):e029846.
8. Mol A. The logic of care: Health and the problem of patient choice: Routledge; 2008.
9. BMA. Pressures in general practice data analysis 2023 [Available from: https://www.bma.org.uk/advice-and-support/nhs-delivery-and-workforce/pressures/pressures-in-general-practice-data-analysis.
10. Cox J, Clark C, Sanders T. Rural healthcare: CRC Press; 2023.
11. Beech J, Fraser C, Gardner T, Buzelli L, Williamson S, Alderwick H. Stressed and overworked: what the Commonwealth Fund's 2022 International Health Policy Survey of Primary Care Physicians in 10 Countries means for the UK. Health Foundation. Mar 2023.

12. NHS. Appointments in General Practice, October 2023 [Available from: https://digital.nhs.uk/data-and-information/publications/statistical/appointments-in-general-practice/october-2023#.

13. Hobbs FDR, Bankhead C, Mukhtar T, Stevens S, Perera-Salazar R, Holt T, et al. Clinical workload in UK primary care: a retrospective analysis of 100 million consultations in England, 2007-14. Lancet. 2016;387(10035):2323-30.

14. Sinnott C, Moxey JM, Marjanovic S, Leach B, Hocking L, Ball S, et al. Identifying how GPs spend their time and the obstacles they face: a mixed-methods study. British Journal of General Practice. 2022;72(715):e148-e60.

15. Khan N. ‘Hidden’no more: the real work of the ‘part-time’GPs working full-time hours. British Journal of General Practice. 2023;73(726):26-7.

16. Freeman G, Hughes, J. Continuity of care and the patient experience 2010 [Available from: https://www.kingsfund.org.uk/sites/default/files/field/field_document/continuity-care-patient-experience-gp-inquiry-research-paper-mar11.pdf.

17. Sandvik H, Hetlevik, O, Blinkenberg, J, Hunskaar, S. Continuity in general practice as predictor of mortality, acute hospitalisation, and use of out-of-hours care: a registry-based observational study in Norway. British Journal of General Practice. 2022;72(715).

18. Kajaria-Montag H, Freeman M, Scholtes S. Continuity of care increases physician productivity in primary care: INSEAD; 2022.

19. Laurant M, Harmsen M, Wollersheim H, Grol R, Faber M, Sibbald B. The impact of nonphysician clinicians. Medical Care Research and Review. 2009;66(6_suppl):36S-89S.

20. Cadogan SL, Browne JP, Bradley CP, Fitzgerald AP, Cahill MR. Physician and practice characteristics associated with immunoglobulin test ordering. Family Practice. 2018;35(1):41-6.

21. Morgan S, Henderson KM, Tapley A, Scott J, Van Driel ML, Spike NA, et al. Pathology test-ordering behaviour of Australian general practice trainees: a cross-sectional analysis. International Journal for Quality in Health Care. 2015;27(6):528-35.

22. Grigoroglou C, Walshe K, Kontopantelis E, Ferguson J, Stringer G, Ashcroft DM, et al. Locum doctor use in English general practice: analysis of routinely collected workforce data 2017–2020. British journal of general practice. 2022;72(715):e108-e17.

23. Nussbaum C, Massou E, Fisher R, Morciano M, Harmer R, Ford J. Inequalities in the distribution of the general practice workforce in England: a practice-level longitudinal analysis. BJGP Open. 2021;5(5).

24. Wong G, Greenhalgh T, Westhorp G, Buckingham J, Pawson R. RAMESES publication standards: realist syntheses. BMC Med. 2013;11:21.

25. Pawson R. Evidence-Based Policy: A Realist Perspective London: SAGE Publications.; 2006.

26. Marchand C, Peckham S. Addressing the crisis of GP recruitment and retention: a systematic review. Br J Gen Pract. 2017;67(657):e227-e37.

27. Andah E, Essang B, Friend C, Greenley S, Harvey K, Spears M, et al. Understanding the impact of professional motivation on the workforce crisis in medicine: a rapid review. BJGP Open. 2021;5(2).

28. Verma P, Ford JA, Stuart A, Howe A, Everington S, Steel N. A systematic review of strategies to recruit and retain primary care doctors. BMC Health Serv Res. 2016;16:126.

29. Lachish S, Svirko E, Goldacre MJ, Lambert T. Factors associated with less-than-full-time working in medical practice: results of surveys of five cohorts of UK doctors, 10 years after graduation. Human resources for health. 2016;14:1-11.

30. Fletcher E, Abel GA, Anderson R, Richards SH, Salisbury C, Dean SG, et al. Quitting patient care and career break intentions among general practitioners in South West England: findings of a census survey of general practitioners. BMJ open. 2017;7(4):e015853.

31. Dale J, Potter R, Owen K, Leach J. The general practitioner workforce crisis in England: a qualitative study of how appraisal and revalidation are contributing to intentions to leave practice. BMC family practice. 2016;17:1-7.

32. Doran N, Fox F, Rodham K, Taylor G, Harris M. Lost to the NHS: a mixed methods study of why GPs leave practice early in England. British journal of general practice. 2016;66(643):e128-e35.

33. Smith F, Goldacre MJ, Lambert TW. Retirement ages of senior UK doctors: national surveys of the medical graduates of 1974 and 1977. BMJ open. 2018;8(6):e022475.

34. Campbell JL, Fletcher E, Abel G, Anderson R, Chilvers R, Dean SG, et al. Policies and strategies to retain and support the return of experienced GPs in direct patient care: the ReGROUP mixed-methods study. 2019;7:1-288.

35. Park S, Berlin A. 'Divided we fail': understanding inter-relations and the role of the generalist. British Journal of General Practice. 2012;62(604):596-8.

36. Bansal A, Greenley S, Mitchell C, Park S, Shearn K, Reeve J. Optimising planned medical education strategies to develop learners' person-centredness: A realist review. Medical Education. 2022;56(5):489-503.

37. Graham A. Written evidence submitted by Dr Anna Graham PhD MRCP MRCGP (FGP0130) 2021 [Available from: https://committees.parliament.uk/writtenevidence/41518/pdf/.

38. Pereira Gray DJ, Sidaway-Lee K, White E, Thorne A, Evans PH. Continuity of care with doctors-a matter of life and death? A systematic review of continuity of care and mortality. BMJ Open. 2018;8(6):e021161.

39. Abrams R, Wong G, Mahtani KR, Tierney S, Boylan A-M, Roberts N, et al. Delegating home visits in general practice: a realist review on the impact on GP workload and patient care. British Journal of General Practice. 2020;70(695):e412-e20.

40. Bienkowska-Gibbs T, King S, Saunders C, Henham M. New organisational models of primary care to meet the future needs of the NHS: a brief overview of recent reports. RAND EUROPE 2015 2015.

41. Wall EM. Continuity of care and family medicine: definition, determinants, and relationship to outcome. J Fam Pract. 1981;13(5):655-64.

42. Haggerty JL, Reid RJ, Freeman GK, Starfield BH, Adair CE, McKendry R. Continuity of care: a multidisciplinary review. BMJ. 2003;327(7425):1219-21.

43. Pereira A. The question is: to GP or not to GP? : RCGP; 2023 [Available from: https://www.rcgp.org.uk/Blog/To-gp-or-not-to-gp.

44. Papoutsi C, Mattick K, Pearson M, Brennan N, Briscoe S, Wong G. Interventions to improve antimicrobial prescribing of doctors in training (IMPACT): a realist review. 2021.

45. Jones R. General practice in the years ahead: relationships will matter more than ever. British Journal of General Practice; 2021. p. 4-5.

46. Jefferson L, Holmes M. GP workforce crisis: what can we do now? : British Journal of General Practice; 2022. p. 206-7.

47. Razai MS, Majeed A. General practice in England: the current crisis, opportunities, and challenges. The Journal of Ambulatory Care Management. 2022;45(2):135-9.

For peer review only

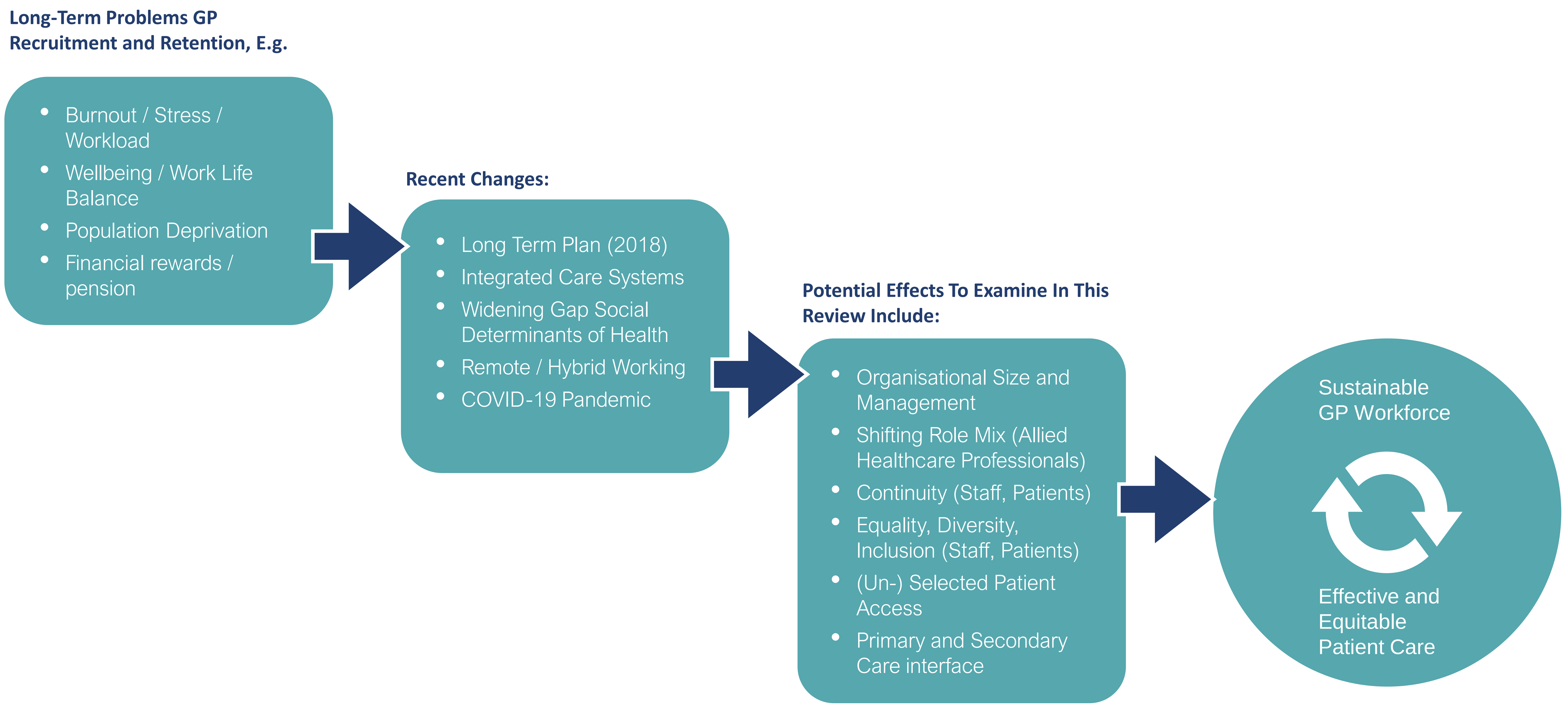
Long-Term Problems GP Recruitment and Retention, E.g.
Burnout / Stress / Workload
Wellbeing / Work Life Balance
Population Deprivation
Financial rewards / pension
Recent Changes:
Long Term Plan (2018)
Integrated Care Systems
Widening Gap Social Determinants of Health
Remote / Hybrid Working
COVID-19 Pandemic
Potential Effects To Examine In This Review Include:
Organisational Size and Management
Shifting Role Mix (Allied Healthcare Professionals)
Continuity (Staff, Patients)
Equality, Diversity, Inclusion (Staff, Patients)
(Un-) Selected Patient Access
Primary and Secondary Care interface
Sustainable GP Workforce
Effective and Equitable Patient Care

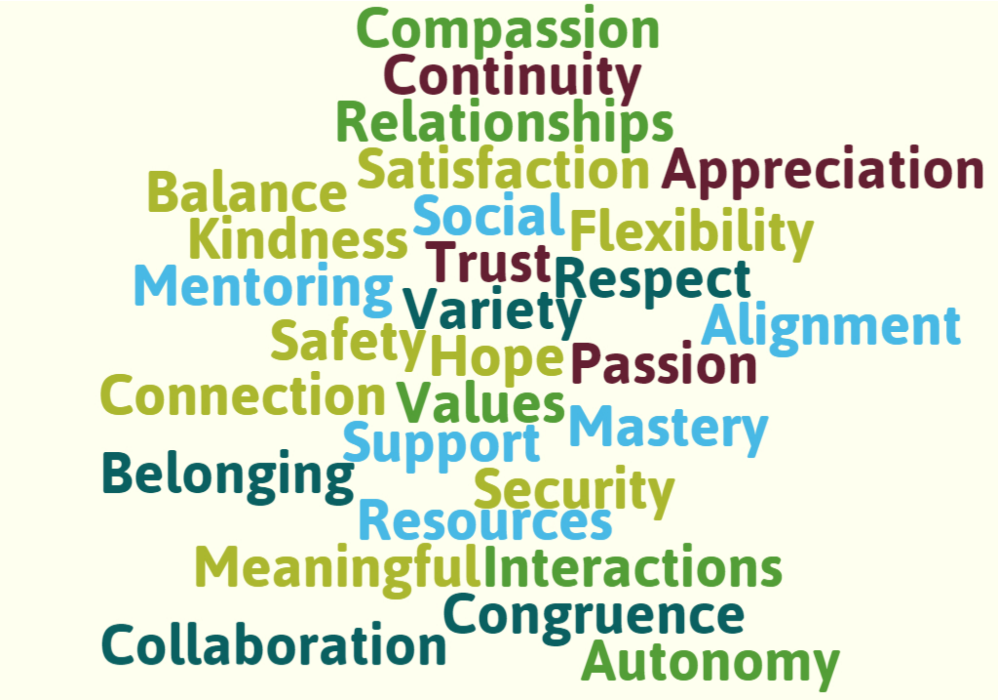


Figure 2: Word cloud visualisation.

361x253mm (72 x 72 DPI)

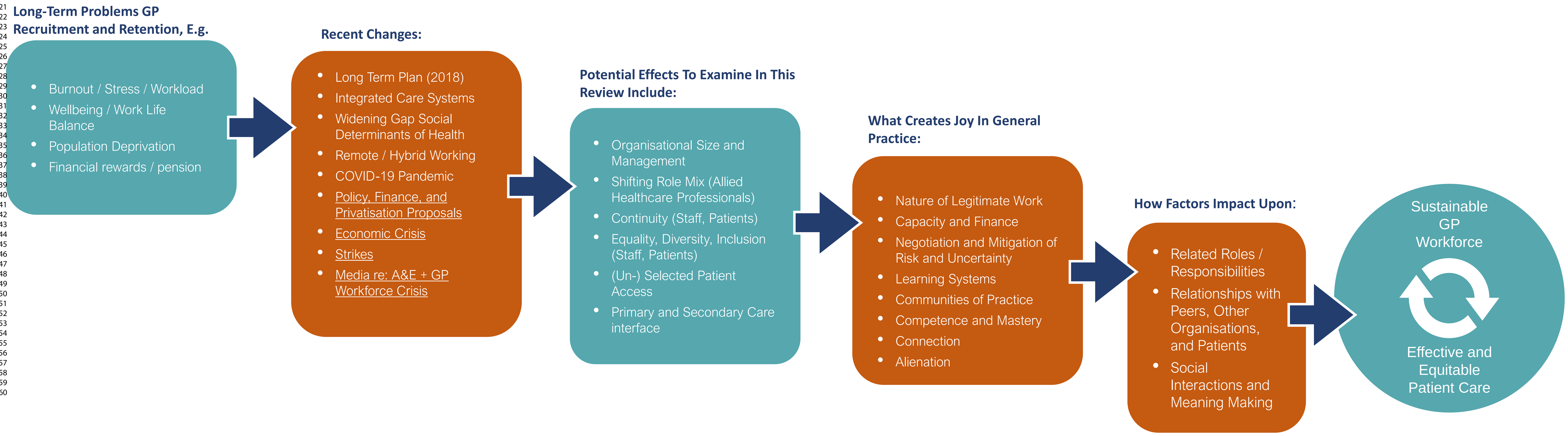
Long-Term Problems GP Recruitment and Retention, E.g.
Burnout / Stress / Workload
Wellbeing / Work Life Balance
Population Deprivation
Financial rewards / pension
Recent Changes:
Long Term Plan (2018)
Integrated Care Systems
Widening Gap Social Determinants of Health
Remote / Hybrid Working
COVID-19 Pandemic
Policy, Finance, and Privatisation Proposals
Economic Crisis
Strikes
Media re: A&E + GP Workforce Crisis
Potential Effects To Examine In This Review Include:
Organisational Size and Management
Shifting Role Mix (Allied Healthcare Professionals)
Continuity (Staff, Patients)
Equality, Diversity, Inclusion (Staff, Patients)
(Un-) Selected Patient Access
Primary and Secondary Care interface
What Creates Joy In General Practice:
Nature of Legitimate Work
Capacity and Finance
Negotiation and Mitigation of Risk and Uncertainty
Learning Systems
Communities of Practice
Competence and Mastery
Connection
Alienation
How Factors Impact Upon:
Related Roles / Responsibilities
Relationships with Peers, Other Organisations, and Patients
Social Interactions and Meaning Making
Sustainable GP Workforce
Effective and Equitable Patient Care

# Supplementary File 1

## Details of the search developed for MEDLINE:

| | | | |
|---|---|---|---|
| **Data Source** | Database | MEDLINE | |
| | Details | MEDLINE® Epub Ahead of Print, In-Process & Other Non-Indexed Citations, Ovid MEDLINE® Daily and Ovid MEDLINE® 1946 to present | |
| | Host | Ovid | |
| | Date searched | 20/04/2023 | |
| | Database Update | Daily update | |
| **Limiters** | English only? | Yes | |
| | Time period searched | 2013 - Present | |
| | Publications types | No limit | |
| | Other | | |
| **Results** | Items found | 504 | |
| | Internal duplicates (within one database) | 6 | |
| | External duplicates (between databases) | 0 | |
| | New | 498 | |
| In Ovid, save your search. Go to **Saved Searches** and click on the eye icon of your SR saved search to **View** your search. Copy and paste your search into **Notepad;** put tabs between the line number and text. Copy and paste into cell **B15**. | **Paste text of search strategy below** | | |
| | **Saved: Sustainable GP V6 FINAL MEDLINE** | | |
| | Medline (Ovid MEDLINE® Epub Ahead of Print, In-Process & Other Non-Indexed Citations, Ovid MEDLINE® Daily and Ovid MEDLINE®) 1946 to present | | |
| | | | |
| | 1 | general practi*.ti,ab,kw. | 92437 |
| | 2 | *General Practitioners/ | 8488 |
| | 3 | exp *General Practice/ | 51683 |
| | 4 | or/1-3 | 121611 |
| | 5 | exp *Work/ | 43272 |
| | 6 | exp *Workplace/ | 14,019 |
| | 7 | *Job Satisfaction/ | 12818 |
| | 8 | exp *Occupational Stress/ | 16249 |
| | 9 | exp *"Personnel Staffing and Scheduling"/ | 24174 |
| | 10 | (work* or staffing or career* or job*).ti,ab,kw. | 2041372 |
| | 11 | (stress* or burn out or burnout).ti,ab,kw. | 1066414 |
| | 12 | or/5-11 | 3013398 |
| | 13 | exp *Workforce/ | 7820 |
| | 14 | *Retirement/ | 6784 |
| | 15 | *Personnel Turnover/ | 3003 |
| | 16 | *Work Engagement/ | 559 |
| | 17 | *Career Mobility/ | 6434 |
| | 18 | (workforce* or staff* level*).ti,ab,kw. | 37788 |
| | 19 | (part?time* or "less than full time" or LTFT).ti,ab,kw. | 239 |
| | 20 | (retire* or retention* or retain* or sustain* or stability or turnover).ti,ab,kw. | 1509240 |
| | 21 | (work* adj1 pattern*).ti,ab,kw. | 1433 |

| | | |
|---|---|---|
| 22 | (flexible* adj1 work*).ti,ab,kw. | 1028 |
| 23 | ((reason* or rationale* or decision* or intention*) adj1 (leav* or stay*)).ti,ab,kw. | 313 |
| 24 | (career* adj1 (break* or end*)).ti,ab,kw. | 312 |
| 25 | ((participat* or engag*) adj1 (work* or job*)).ti,ab,kw. | 6597 |
| 26 | or/13-25 | 1560462 |
| 27 | 4 and 12 and 26 | 2544 |
| 28 | exp United Kingdom/ | 389181 |
| 29 | (national health service* or nhs*).ti,ab,in. | 265557 |
| 30 | (english not ((published or publication* or translat* or written or language* or speak* or literature* or citation*) adj5 english)).ti,ab. | 48488 |
| 31 | (gb or "g.b." or britain* or (british* not "british columbia") or uk or "u.k." or united kingdom* or (england* not "new england") or northern ireland* or northern irish* or scotland* or scottish* or ((wales or "south wales") not "new south wales") or welsh*).ti,ab,jw,in. | 2421175 |
| 32 | (bath or "bath's" or ((birmingham not alabama*) or ("birmingham's" not alabama*) or bradford or "bradford's" or brighton or "brighton's" or bristol or "bristol's" or carlisle* or "carlisle's" or (cambridge not (massachusetts* or boston* or harvard*)) or ("cambridge's" not (massachusetts* or boston* or harvard*)) or (canterbury not zealand*) or ("canterbury's" not zealand*) or chelmsford or "chelmsford's" or chester or "chester's" or chichester or "chichester's" or coventry or "coventry's" or derby or "derby's" or (durham not (carolina* or nc)) or ("durham's" not (carolina* or nc)) or ely or "ely's" or exeter or "exeter's" or gloucester or "gloucester's" or hereford or "hereford's" or hull or "hull's" or lancaster or "lancaster's" or leeds* or leicester or "leicester's" or (lincoln not nebraska*) or ("lincoln's" not nebraska*) or (liverpool not (new south wales* or nsw)) or ("liverpool's" not (new south wales* or nsw)) or ((london not (ontario* or ont or toronto*)) or ("london's" not (ontario* or ont or toronto)) or manchester or "manchester's" or (newcastle not (new south wales* or nsw)) or ("newcastle's" not (new south wales* or nsw)) or norwich or "norwich's" or nottingham or "nottingham's" or oxford or "oxford's" or peterborough or "peterborough's" or plymouth or "plymouth's" or portsmouth or "portsmouth's" or preston or "preston's" or ripon or "ripon's" or salford or "salford's" or salisbury or "salisbury's" or sheffield or "sheffield's" or southampton or "southampton's" or st ablans or stoke or "stoke's" or sunderland or "sunderland's" or truro or "truro's" or wakefield or "wakefield's" or wells or westminster or "westminster's" or winchester or "winchester's" or wolverhampton or "wolverhampton's" or (worcester not (massachusetts* or boston* or harvard*)) or ("worcester's" not (massachusetts* or boston* or harvard*)) or (york not ("new york" or ny or ontario* or ont or toronto*)) or ("york's" not ("new york" or ny or ontario* or ont or toronto*))))).ti,ab,in. | 1720005 |
| 33 | (bangor* or "bangor's" or cardiff or "cardiff's" or newport or "newport's" or st asaph or "st asaph's" or st davids or swansea or "swansea's").ti,ab,in. | 69203 |
| 34 | (aberdeen or "aberdeen's" or dundee or "dundee's" or edinburgh or "edinburgh's" or glasgow or "glasgow's" or inverness or (perth not australia*) or | 253603 |
| 35 | "newry's").ti,ab,in. | 33231 |
| 36 | or/28-35 | 3037247 |
| 37 | (exp africa/ or exp americas/ or exp antarctic regions/ or exp arctic regions/ or exp asia/ or exp oceania/) not (exp United Kingdom/ or europe/) | 3308914 |
| 38 | 36 not 37 | 2875391 |
| 39 | 27 and 38 | 793 |
| 40 | limit 39 to (english language and yr="2013 -Current") | 504 |
